# Effects of time-restricted eating with different eating windows on human metabolic health: pooled analysis of existing cohorts

**DOI:** 10.1186/s13098-023-01190-y

**Published:** 2023-10-24

**Authors:** Zhongbiao Nie, Jiaming Xu, Yinchu Cheng, Zhihong Li, Ran Zhang, Wentao Zhang, Libo Zhao

**Affiliations:** 1 Department of Pharmacy, Peking University Third Hospital, Beijing, 100191 China; 2 Department of Pharmacy, Shanxi Bethune Hospital, Shanxi Academy of Medical Sciences, Tongji Shanxi Hospital, Third Hospital of Shanxi Medical University, Taiyuan, 030032 China; 3 Nephrology Department, Affiliated Hospital of Shanxi University of Chinese Medicine, Taiyuan, 030036 China; 4 Pharmacy Department, Beijing hospital of Integrated traditional Chinese and Western Medicine, Beijing, 100039 China

**Keywords:** Time-restricted eating, Eating windows, Metabolic health, Pooled analysis, Existing cohorts

## Abstract

**Background:**

Time-restricted eating (TRE), a feasible form of intermittent fasting, has been proven to benefit metabolic health in animal models and humans. To our knowledge, specific guidance on the appropriate period for eating during TRE has not yet been promoted. Therefore, to compare and assess the relative effectiveness estimates and rankings of TRE with different eating windows on human metabolic health, we conducted a systematic review and network meta-analysis (NMA).

**Method:**

PubMed, EMBASE and the Cochrane Library were searched for randomized controlled trials that compared different eating windows on human metabolic health for adults. A Bayesian NMA was used to compare direct and indirect effects to determine the best different eating windows, and scientific evidence using GRADE.

**Results:**

Twenty-seven RCTs comparing TRE with different eating windows on human metabolic health were reviewed, and all were included in the NMA. Compared with the normal diet group (non-TRE), the TRE group has certain benefits in reducing weight and fasting insulin. In terms of reducing fasting insulin, the 18:6 group (eating time = 6 h) was better than the 14:10 group (eating time = 10 h) and 16:8 group (eating time = 8 h) (P < 0.05); The < 6 group (eating time < 6 h) was better than the 14:10 group (P < 0.05). In terms of reducing fasting glucose, the < 6 group was better than the 14:10 group (P < 0.05). There were no statistical variations in weight, HDL, TG, and LDL across the different modes of TRE (P > 0.05).

**Conclusions:**

Our research showed that no particular metabolic advantages of various eating windows were found. Therefore, our results suggested that different eating windows could promote similar benefits for metabolic parameters.

**Supplementary Information:**

The online version contains supplementary material available at 10.1186/s13098-023-01190-y.

## Introduction

Metabolic health is determined by long-term dietary patterns [1]. More recently, fasting regimens, such as caloric restriction and intermittent fasting (IF), have been shown to reduce body mass, serum insulin concentration, blood pressure (BP), and inflammation, and to improve insulin sensitivity and the lipid profile, thereby reducing the risk for metabolic disease [2–5]. Time-restricted eating (TRE) is a form of IF that involves restricting the daily window for food consumption to a period of 3 to 12 h [6, 7]. Several meta-analyses suggest that TRE can effectively manage weight and enhance metabolic health [8–10]. It has been shown to increase high-density lipoprotein cholesterol (HDL-C), decrease blood pressure, insulin resistance, and circulating levels of triglyceride (TG), total cholesterol (TC), and low-density lipoprotein cholesterol (LDL-C). Additionally, it has been demonstrated to decrease body weight, fat mass, and waist circumference (WC) [10] TRE has developed into a desirable and straightforward lifestyle intervention [6, 9, 11].

To date, numerous TRE (4–10 h eating sessions) pilot studies have been carried out. Unexpectedly, the outcomes of TRE in humans seem to be influenced by the time of day the eating window is [12–16]. Although two of the most popular forms of TRE followed by the general public are 4-h TRE (a.k.a. “The Warrior Diet”) and 8-h TRE (a.k.a. “The 16:8 Diet”),, there have been many patterns of TRE in recent years. Tingting Che et al [17] have explored the effect of 10-h TRE on type 2 diabetes, 10-h restricted eating enhances the quality of life, increases blood glucose and insulin sensitivity, causes weight reduction, lowers the required dosage of hypoglycaemic medications, and results in weight loss. Additionally, it can improve cardiovascular health by lowering atherosclerotic TC levels. Cienfuegos et al [18] examined the impact of two well-liked TRE schedules (4-h and 6-h) and similar reductions in body weight, energy consumption, insulin resistance, and oxidative stress were observed with both diets.

To our knowledge, previous research suggests that the precise time window for eating may have an impact on the effects of TRE; however, the ideal meal windows for TRE have not yet been identified. Other researchers have been perplexed by the diversity and inconsistency of TRE findings due to the many eating windows. In this study, to assess and contrast the effects of various TRE eating windows on weight reduction and other metabolic-related parameters in adults, we set out to perform a network meta-analysis and systematic review of RCTs.

## Method

### Registration

Following the international Preferred Reporting Items for Systematic Reviews and Meta-Analyses for Network Meta-Analyses (PRISMA-NMA) principles, this work is a systematic evaluation of the literature that is descriptive [19]. It was registered with the PROSPERO (CRD42023388830).

### Search Strategy

The literature search was done by the PRISMA-NMA suggested protocol (Supplementary Materials Table S1). Two researchers extracted the data (Nie and Xu). Two reviewers (Nie and Xu) improved the data extraction tables before the data extraction. Using the same search keywords on the same day, Nie and Xu tested for correctness by cross-referencing results from citation databases like PubMed. Nie and Xu independently extracted data using the established data extraction tables. Disparities were clarified by a discussion with a third investigator (Zhao). In addition, we conduct a “snowball search” to add other articles. We also looked up gray literature on Google. The searches were conducted in any language.

### Study selection

We included studies with the following criteria: (1) an adult population; (2) RCT studies; (3) TRE intervention; (4) at least a two-week follow-up period; (5) studies reporting results that included at least one of the following measurements: body weight, body mass index (BMI), fasting glucose, glycosylated hemoglobin (HbA1c), systolic blood pressure (SBP), diastolic blood pressure (DBP), TC, HDL, low-density lipoprotein (LDL), TG, or fasting insulin; (6) trials that have been published.

**Fig. 1:**
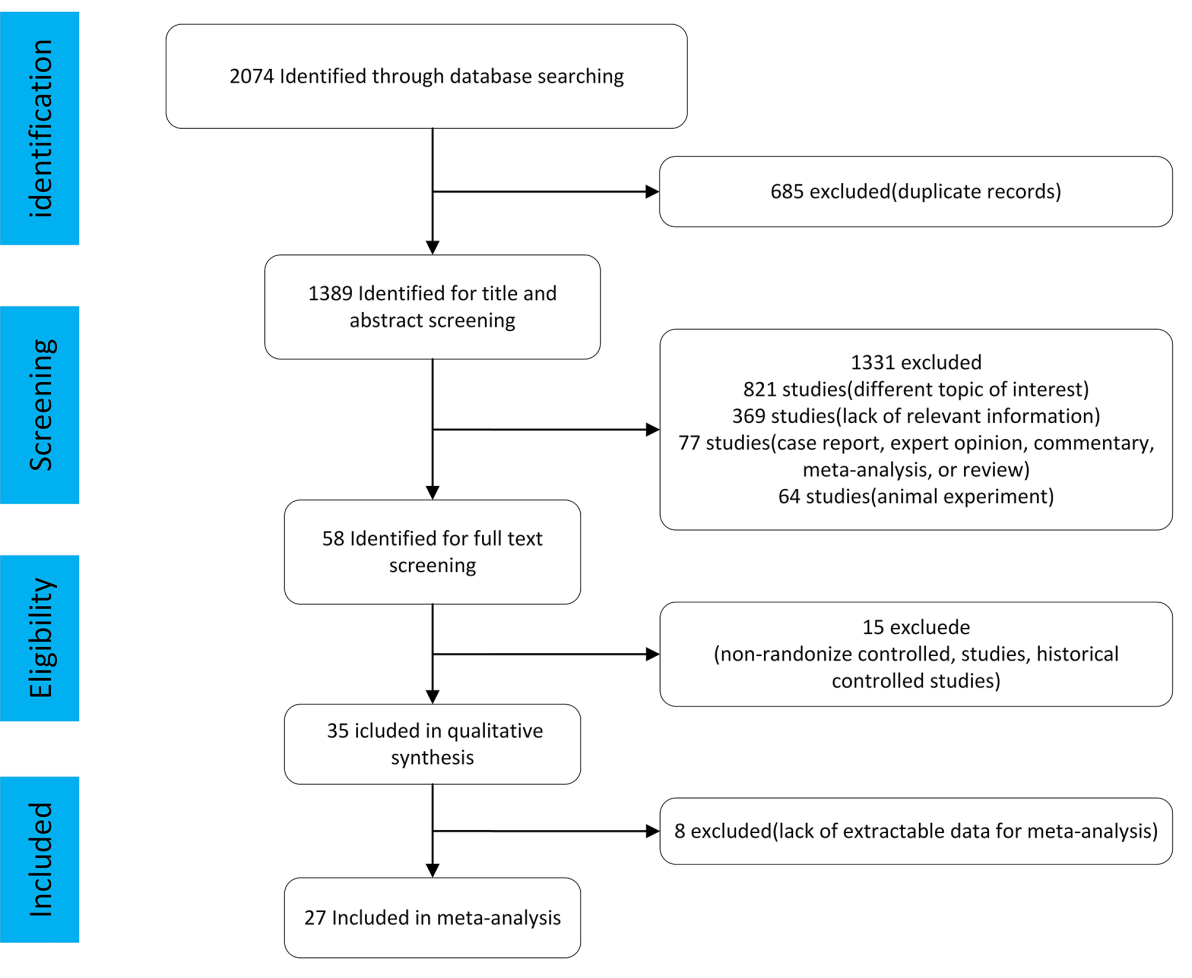
Search strategy

We excluded studies with the following criteria: (1) duplications of the studies that had already been searched; (2) non-RCTs, and nonoriginal articles such as review articles, editorials, case reports, or letters; (3) animal studies; (4) Ramadan studies (an Islamic tradition that requires people to eat only after sunset). Its effects on human metabolic health are controversial; (5) other studies that did not meet the aforementioned inclusion criteria. Based on these criteria, we ultimately included 27 papers for analysis after excluding 2047 research (Fig. 1).

### TRE categories

Five categories were used to classify the TRE interventions for the included RCTs according to different eating windows. Table 1 gives a thorough explanation of each TRE category.

**Table 1:** Explanation of the TRE category

Type of TRE	Abbreviation	Definition
Eating time < 6 h	< 6	①eating only between 15:00 and 18:00; ② restricted their daily eating duration by 3 h.
Eating time = 6 h	18:6	① eating only between 13:00 and 19:00; ②eating between 8:00 and 14:00.
Eating time = 8 h	16:8	① eating between 7:00 and 15:00; ② eating between 8:00 and 16:00; ③ eating between 10:00 and 18:00; ④ eating between 12:00 and 20:00; ⑤ eating between 11:00 and 20:00, there should be no more than 8 h of eating.
Eating time = 10 h	14:10	① eat only during a window of 10 h, starting within 3 h of waking; ② eating between 10:00 and 20:00; ③eating between 8:00 to 18:00.
Eating time = 12 h	= 12	eat only in a 12-hour window.
No TRE	Control group	Normal diet (non-TRE).

### Data extraction

The following items were independently extracted by two experienced authors (Nie and Xu): TRE Regimen, Study design, sample size, geographic region, participants, study duration, weight, age, and sex. Baseline information was taken for our continuous variables in both the TRE and control groups (Table 2).

**Table 2:** Experimented with details of the 26 studies selected in the review

TRE Regimen	Study Design	Study[Ref.](Year)	Sample size(TRE/CON)	Geographic region	participants	Study duration	Weight(kg) TRE/CON	Age(year) TRE/CON	Sex (men/women) TRE/CON
16:8	RCT	Kord-Varkaneh H et al.(2022) [43]	22/23	USA	non-alcoholic fatty liver disease	12 weeks	83.75 ± 12.71/89.33 ± 18.47	41.36 ± 10.5 /44.17 ± 4.9	12(10)/15(8)
16:8	RCT	Moro T et al. (2021) [44]	10/10	ITALY	healthy adults	12 months	83.22 ± 5.92/84.64 ± 5.76		
16:8	RCT	Kotarsky C J et al.(2021) [45]	11/10	USA	overweight and obese adults	8 weeks	82 ± 3/83 ±3	83 ± 3/82 ± 3	
16:8	RCT	He M et al.(2022) [46]	44/47	China	visceral fat	3 months	84.3 ± 2.2/84.7 ± 2.0	43.0 ± 1.4/41.3 ± 1.4	30(14)/35(12)
16:8	PRE–POST randomized design	Brady A J et al. (2021) [47]	12/11	IRELAND	male Middle- and Long-Distance Runners	8 weeks	73.13 ± 6.06/72.17 ± 6.68	35.9 ± 8.6/39.9 ± 3.0	
16:8	RCT	Queiroz J.D.N et al. (2022) [11]	13/13	Brazil	adults with overweight and obesity	8 weeks	83·4 ± 10·6/81·4 ± 13·1	26 ± 4/33 ± 6	2(11)/2(11)
16:8	RCT	Chow L S et al. (2020) [24]	11/9	USA	humans who are Overweight	12 weeks	95.2 ± 22.6/100.9 ± 28.1	46.5 ± 12.4/44.2 ± 12.3	2(9)/1(8)
16:8	RCT	Lin Y J et al. (2021) [48]	30/33	Taiwan	middle-aged women	8 weeks	65.9 ± 9.7/65.8 ± 8.8	50.1 ± 7.5/54.2 ± 7.9	
16:8	RCT	Moro T et al.(2020) [14]	8/8	Italy	elite cyclists	4 weeks	67.04 ± 5.03/72.27 ± 6.24	19.38 ± 2.39/19.38 ± 1.60	
16:8	RCT	Cai H et al.(2019) [49]	95/79	China	non-alcoholic fatty liver disease	12 weeks	74.98 ± 8.02/72.94 ± 8.00	33.56 ± 6.23/34.5 ± 6.96	60(35)/66(13)
16:8	RCT	Tinsley G M et al. (2019) [16]	8/9	Italy	active females	8 weeks	63.8 ± 8.5/64.6 ± 8.8	23.3 ± 1.5/22.6 ± 2.7	
16:8	RCT	Lowe D A et al.(2020) [50]	59/57	USA	adults with Overweight and Obesity	12 weeks	99.3 ± 16.9/99.1 ± 15.1	46.8 ± 10.8/46.1 ± 10.3	35(24)/35(22)
16:8	RCT	Jamshed H et al. (2022) [51]	45/45	USA	adults With Obesity	14 weeks	112.3 ± 20.1/105.3 ± 20.7	43 ± 11/43 ± 10	8(37) 10(35)
16:8	RCT	Liu D et al. (2022) [41]	69/70	China	obesity	12 months		31.6 ± 9.3/32.2 ± 8.8	
16:8	RCT	Xie Z et al.(2022) [40]	26/28	China	healthy volunteers without obesity	5 weeks	61.1 ± 8.8/61.2 ± 9.9	28.68 ± 9.707/33.57 ± 11.6	9(17)/4(24)
16:8	RCT	Bei-ni Lao et al.(2023) [52]	13/14	China	overweight and obese patients with chronic kidney disease stages 3–4	12 weeks	51.8 ± 7.7/52.5 ± 11.3	51.8 ± 7.7/52.5 ± 11.3	7(6)/7(7)
14:10	RCT	Haganes K L et al.(2022) [53]	33/33	Norway	women with overweight/obesity	7 weeks	91.0 ± 10.8/95.0 ± 11.2	36.2*5.9/36.4*6.2	
14:10	RCT	Manoogian E et al.(2022) [54]	70/67	USA	24-h shift workers	12 weeks	108.07 ± 1.75/107.1 ± 5.35	41.07 ± 8.71/39.61 ± 9.39	65(5)/60(7)
14:10	RCT	Che T et al.(2021) [17]	54/50	China	overweight patients with type 2 diabetes	12 weeks	75.06 ± 4.42/74.68 ± 4.35	48.21 ± 9.32/48.78 ± 9.56	25(29)/24(26)
14:10	Randomized crossover trial	Andriessen C et al.(2022) [55]	7/7	Netherlands	adults with type 2 diabetes but does not improve insulin sensitivity	3 weeks		67.5 ± 5.2/66.9 ± 5.1	7(0)/7(0)
14:10	RCT	Thomas E A et al.(2021) [56]	32/31	USA	adults with obesity	39 weeks	96.1 ± 18.1/93.4 ± 18.4	38.3 ± 7.9/37.8 ± 7.8	7(35)/5(31)
12:12	RCT	Phillips N.E et al.(2021) [57]	25/20	Switzerland	community-Based Adults	6 months	79.6 ± 15.9/77.5 ± 13.8		
12:12	RCT	de Oliveira M P I et al.(2021) [11]	31/27	Brasil	low-income women with obesity	12-months	81.25 ± 13.51/80.25 ± 9.40		
21:3	RCT	Rona A et al.(2018) [58]	7/6	UK	free-living human	10 weeks	86.2 ± 5.2/77.8 ± 7.6	47 ± 3/45 ± 4	1(6)/0(6)
20:4 18:6	RCT	Cienfuegos S et al.(2020) [18]	19/19 20/19	USA	adults with obesity	8 weeks	100 ± 4/94 ± 3 99 ± 4/94 ± 3	47 ± 2/45 ± 2 47 ± 3/45 ± 2	2(17)/2(17) 3(17)/2(17)
18:6	RCT	Mayra S T et al.(2022) [59]	8/10	USA	college students	4 weeks	68.3 ± 11.3/67.6 ± 10.6	25.1 ± 4.1/21.8 ± 3.8	1(7)/2(8)
18:6	RCT	Sutton E F et al(2018) [25]	5/7	USA	men with Prediabetes	5weeks			

### Outcome measures

The primary outcome measure was body weight. The secondary outcomes included BMI, TC, HbA1c, fasting glucose, SBP, DBP, LDL, HDL, and TG.

### Data analyses and statistical analysis

The geometry of the various exercise interventions was described and presented in network plots using STATA’s “network plot” and “publication bias” functions. If SDs were not reported, we calculated them from standard errors (SEs) or confidence intervals (CIs). A Bayesian hierarchical model (binomial modeling with logit link function) was used for the analysis, along with a Markov chain Monte Carlo method. We performed 5000 iterations and 3000 adaptations with a 10-thinning factor. A possible scale reduction factor was used to evaluate the convergence of the results. The Gelman-Rubin diagrams displayed the model’s convergence diagnostics (Figures S1). From the direct estimates with a common arm, the indirect estimates were calculated using the consistency equation. Credible intervals (CrIs) were indicated for the results. For all the included treatment nodes, rank probabilities, the preferred order of therapeutic success, were determined based on the distribution of CrIs. The surface under the cumulative ranking curve (SUCRA) score was used to establish a treatment hierarchy after we calculated the cumulative probabilities for each intervention at each conceivable rank [20, 21]. With the aid of the “gemtc” package (version 0.8-7, Github.com, GitHub, Inc, San Francisco, CA), the statistical analysis was carried out in R (The R Foundation for Statistical Computing, Vienna, Austria). We calculated κ statistics to assess the agreement between the two investigators for the assessment of methodological quality.

### Assessment of Study Quality and Publication Bias

To evaluate the risk of bias in the included RCTs, we used a modified Cochrane risk of bias tool. The biases included reporting bias, attrition bias, detection bias, performance bias, and selection bias. The specific items were related to the following 6 aspects: the creation of random sequences, concealment of allocations, participant and researcher blinding, insufficient outcome data, selective reporting, and additional bias. Egger’s test was used to measure publication bias and funnel plot asymmetry was used to evaluate it. The Grading of Recommendations Assessment, Development and Evaluation (GRADE) approach to rating the quality of evidence for network meta-analysis was used for this study. The GRADE four-step approach was used. First, the direct and indirect treatment estimates for each comparison were determined. Second, the quality of each direct and indirect effect estimate was rated. Since all included studies are RCTs, all trials started with a quality rating of high-quality evidence. Studies were rated down for the following reasons: (a) risk of bias based on randomization, blinding, and attrition; (b) inconsistency based on heterogeneity of effect estimates across trials; (c) indirectness; (d) imprecision; and (e) publication bias [22].

## Results

### Study characteristics

The literature search turned up 2074 publications, including clinical trials, literature reviews, and other pertinent work (PubMed: 1861, EMBASE: 765, Cochrane Library: 448). 685 duplicate articles were eliminated, leaving 1389 articles, of which 27 were ultimately chosen. Thorough illustration of the procedure is described in Fig. 1. Explanation of the TRE category is described in Table 1. The research involved participants from America (12), Europe (8), and Asia (7). This study comprised 1531 participants in total. There were 9 studies including healthy persons and 18 research involving subjects with metabolic abnormalities (overweight/obesity, prediabetes, metabolic syndrome, and non-alcoholic fatty liver disease).

### Quality Assessment

**Fig. 2:**
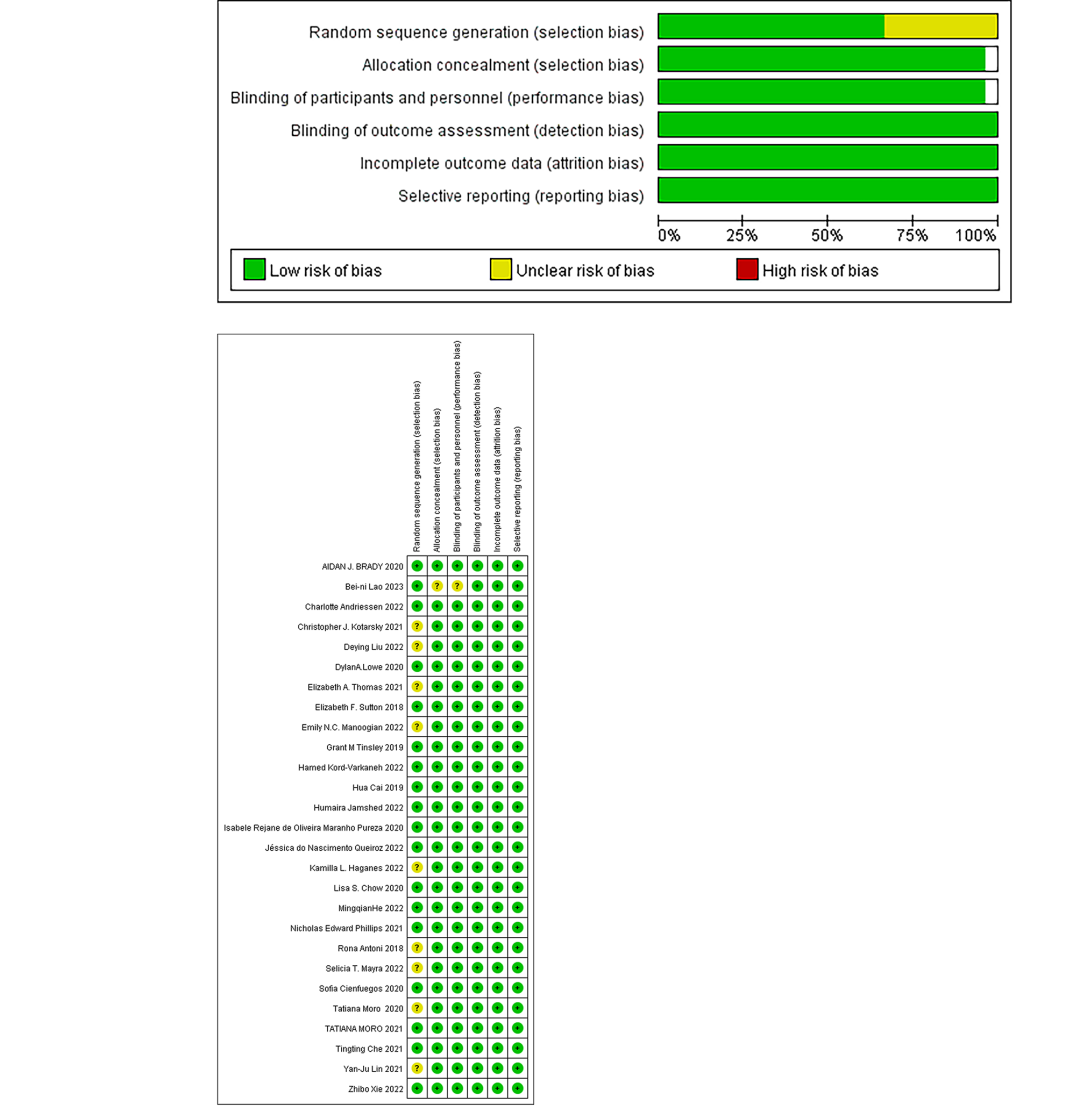
Risk-of-bias assessment in the studies included in the meta-analysis

Figure 2 A and 2B report the bias risk for the RCTs. A low risk of bias existed in 17 studies. Due to a lack of information regarding the randomization procedure, 9 RCTs exhibited some bias concerns. Despite being a randomized experiment, the participants’ knowledge of the intervention could not be concealed. Therefore, for unblinded experimental research, we all considered it low-risk. The inter-rater reliability for assessment of quality items was 0.59 (P < 0.001). Overall, the methodological quality was moderate.

### Network meta-analysis

**Fig. 3:**
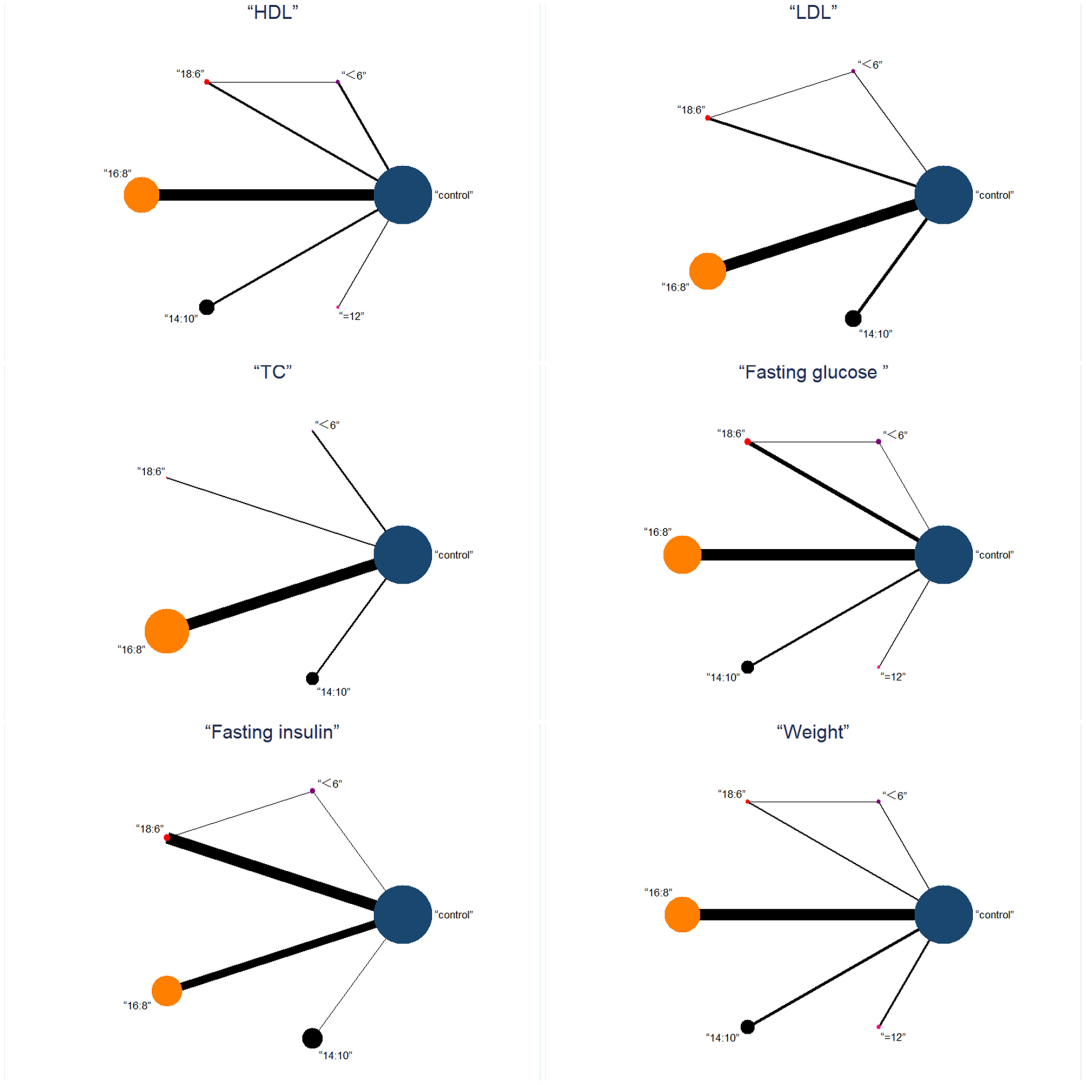
Network meta-analysis maps of the different eating windows with TRE with different eating windows on metabolic health about Weight, Fasting insulin, HDL, LDL, Fasting glucose, and TG. The number of participants in each intervention type is represented by the size of the nodes, and the number of studies used to make the comparison is represented by the thickness of the lines between interventions

Six indicators of metabolic health (weight, fasting insulin, HDL, LDL, fasting glucose, TG) were included in the NMA. The pre-post data for the six outcomes included in the NMA are shown in Table S2. Incorporating these variables into the NMA is impractical due to the minimal number of studies reporting pre-post intervention levels and/or changes in BMI, TC, HbA1c, SBP, and DBP. Therefore, it was infeasible to incorporate them into the NMA (Table S3). NMA maps of the studies on the efficacy of TRE with different eating windows on weight are illustrated in Fig. 3, include weight, fasting insulin, HDL, LDL, fasting glucose, and TG. Table 3 details the complete matrix of results, and Table S4 ranks the likelihood of the measured results having the desired effect according to different eating windows.

**Table 3:** Network meta-analysis matrix of results

Outcome	Comparison of treatments: Mean difference (95% confidence intervals) Effect of intervention in each row compared with intervention in each column					
Weight(Kg)	Control	< 6	18:6	16:8	14:10	= 12
**Control**	1	-1.53(-14.38, 14.83)	-2.95(-16.58, 13.96)	4.56(-3.16, 13.07)	9.54(-1.32, 22.16)	17.76(-11.76, 48.0)
**< 6**		2	-1.34(-18.16, 15.93)	6.12(-11.90, 21.50)	11.08(-8.01, 28.83)	19.13(-14.35, 51.53)
**18:6**			3	7.46(-11.06, 23.29)	12.38(-7.18, 30.65)	20.46(-13.26, 52.82)
**16:8**				4	5.05(-8.96, 19.70)	13.19(-17.42, 44.23)
**14:10**					5	8.16(-23.77, 40.23)
**= 12**						6
**Fasting insulin (µU/mL)**	**Control**	**< 6**	**18:6**	**16:8**	**14:10**	
**Control**	1	-3.13(-5.49, -0.60)	-3.87(-6.31, -1.46)	-0.73(-2.06, 0.44)	0.21(-1.45, 2.00)	
**< 6**		2	-0.74(-3.69, 1.99)	2.40(-0.55, 5.02)	3.34(0.29, 6.29)	
**18:6**			3	3.14(0.32, 5.80)	4.08(1.15, 7.16)	
**16:8**				4	0.94 (-1.044, 3.24)	
**14:10**					5	
**TG (mg/dL)**	**Control**	**< 6**	**18:6**	**16:8**	**14:10**	**= 12**
**Control**	1	-1.68(-14.09, 15.27)	-3.05(-16.29, 14.97)	4.45(-3.07, 12.89)	9.48(-1.40, 21.97)	18.47(-12.35, 47.715)
**< 6**		2	-1.36(-18.30, 15.83)	6.02(-12.18, 21.05)	11.10 (-8.70, 28.37)	19.67(-15.83, 51.03)
**18:6**			3	7.214(-11.84, 23.16)	12.45(-7.65, 30.37)	21.26(-15.07, 53.08)
**16:8**				4	5.17(-8.88, 19.60)	13.84(-18.23, 44.00)
**14:10**					5	8.89(-24.79, 40.02)
**= 12**						6
**LDL (mg/dL)**	**Control**	**< 6**	**18:6**	**16:8**	**14:10**	
**Control**	1	-3.57(-14.16, 5.11)	-5.39(-14.93, 3.35)	-0.32(-4.48, 4.869)	-0.07(-8.74, 8.75)	
**< 6**		2	-1.73(-11.86, 9.06)	3.23(-6.06, 15.34)	3.61(-8.57, 17.20)	
**18:6**			3	5.02(-4.34, 16.39)	5.36(-7.05, 18.12)	
**16:8**				4	0.21(-9.95, 9.60)	
**14:10**					5	
**HDL (mg/dL)**	**Control**	**< 6**	**18:6**	**16:8**	**14:10**	**= 12**
**Control**	1	-2.06(-6.20, 2.05)	1.49(-2.07, 4.94)	0.22(-1.48, 1.89)	1.09(-2.05, 4.28)	1.97(-6.80, 10.66)
**< 6**		2	3.56(-0.98, 8.01)	2.29(-2.21, 6.74)	3.16(-2.01, 8.37)	4.04(-5.62, 13.61)
**18:6**			3	-1.26(-5.10, 2.68)	-0.38(-5.07, 4.41)	0.47(-8.89, 9.86)
**16:8**				4	0.85(-2.68, 4.51)	1.72(-7.20, 10.58)
**14:10**					5	0.85(-8.41, 10.16)
**= 12**						6
**Fasting glucose (mg/dL)**	**Control**	**< 6**	**18:6**	**16:8**	**14:10**	**= 12**
**Control**	1	-5.09(-12.13, 2.13)	-4.08(-10.76, 2.88)	-2.13(-5.63, 1.28)	4.64(-1.56, 11.17)	-4.31(-16.07, 7.29)
**< 6**		2	0.93(-7.42, 9.53)	2.94(-5.15, 10.77)	9.73(0.15, 19.51)	0.71(-12.82, 14.40)
**18:6**			3	1.92(-5.97, 9.42)	8.76(-0.69, 18.04)	-0.26(-13.86, 13.33)
**16:8**				4	6.79(-0.24, 14.29)	-2.20(-14.26, 10.07)
**14:10**					5	-8.99 (-22.54, 4.03)
**= 12**						6

#### Body weight

25 studies contributed to the NMA assessment. Network meta-analysis suggested that there was no statistical difference in comparison between each group in weight (P > 0.05). Moreover, SUCRA analysis findings that the 18:6 group had the highest probability of being best (84.2%), followed by the < 6 group (79%), 14:10 group (66.3%), 16:8 group (46.8%) (Figure S2).

#### Fasting insulin

20 studies contributed to the NMA assessment. Network meta-analysis suggested that the < 6, 18:6 group was significant superior to the control group in reducing fasting insulin [MD_< 6_=-3.13(-5.49, -0.60); MD_18:6_=-3.87(-6.31, -1.46)]; The 18:6 group was better than the 14:10 and 16:8 group [MD_18:6_ vs. _14:10_=3.14(0.32, 5.80); MD_18:6 vs. 16:8_=4.08(1.15, 7.16)]; The < 6 group was better than the 14:10 group [MD_< 6 vs. 14:10_=3.34(0.29, 6.29)]. Moreover, SUCRA analysis found that the 18:6 group had the highest probability of being best (94%), followed by the < 6 group (81%), and 14:10 group (48.7%) (Figure S2).

#### HDL and LDL

23 studies contributed to the NMA assessment. Network meta-analysis suggested that there was no statistical difference in comparison between each group in HDL and LDL (P > 0.05). Moreover, SUCRA analysis in HDL analysis found that the 18:6 group had the highest probability of being best (70.6%), followed by the = 12 group (64.8%), 14:10 group (64.1%) (Figure S2). SUCRA analysis in LDL analysis findings that the 18:6 group had the highest probability of being best (80.7%), followed by the < 6 group (65.9%) (Figure S2).

#### Fasting glucose

22 studies contributed to the NMA assessment. Network meta-analysis suggested that the < 6 group was significantly better than the 14:10 group [MD_< 6 vs. 14:10_=9.73 (0.15, 19.51)]. Moreover, SUCRA analysis findings that the < 6 group had the highest probability of being best (89.7%), followed by the = 12 group (74.6%) (Figure S2).

#### TG

25 studies contributed to the NMA assessment. Network meta-analysis suggested that there was this difference was not statistically significant in comparison between each group in TG. Moreover, SUCRA analysis findings that the 18:6 group had the highest probability of being best (91.1%), followed by the < 6 group (88%), and the 16:8 group (50.8%) (Figure S2).

#### Radar graphic

**Fig. 4:**
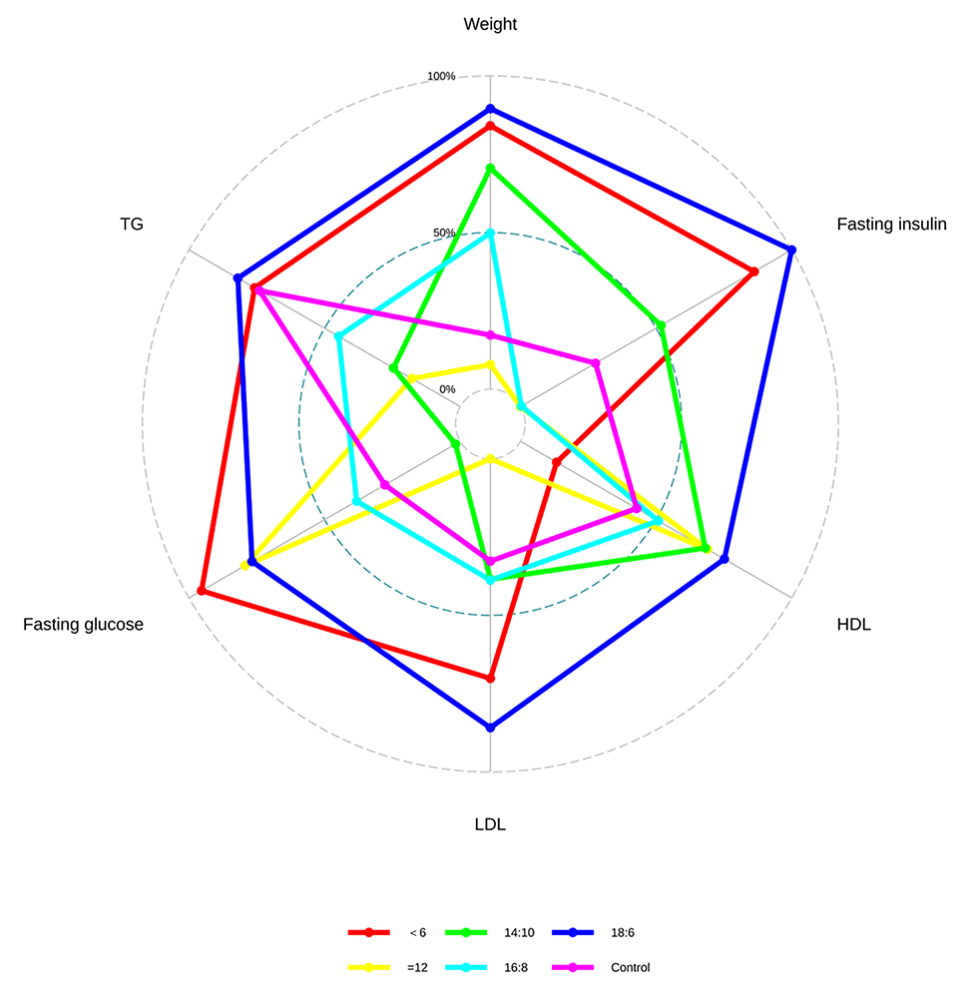
Radar graphic showing different doses for all results. This radar plot’s six angles correspond to the results. The ranking probability is represented by each pentagon in the radar map, and the greater the pentagon, the higher the ranking probability. The SUCRA scores are a frequently used approach to numerically summarize the cumulative rankings, where a therapy receives a score of 1 if it is unquestionably the best and a score of 0 if it is unquestionably the worst

Based on the findings of the SUCRA analysis, we developed a Radar graphic to identify which treatment option may be best for a particular outcome. As shown in Fig. 4, although there is no statistical difference, the 18:6 group near the edge of the radar image for weight, fasting insulin, HDL, LDL, and TG, indicating that it might be the best treatment option for lowering weight, fasting insulin, HDL, LDL, and TG. In terms of fasting glucose, < 6 group near the edge of the radar image, indicating that it might be the best treatment option for lowering fasting glucose.

### Inconsistency and publication bias

It can be seen from Figure S3, the node-split method did not determine loop-closed inconsistency for fasting insulin, HDL, LDL, fasting glucose, and TG, but the weight was accessible for loop-closed inconsistency. A funnel plot with comparison adjustments was developed to assess publication bias. Except for weight and LDL, symmetrical outlines were created for each result, as seen in Figure S4.

### Synthesis of evidence

Table 4 summarizes the details of the GRADE assessment of certainty of the evidence for the primary and secondary outcomes. We conducted a GRADE assessment on 6 outcomes of 27 RCTs. Except HDL provided strong evidence, other outcomes are rated as moderate or low quality are primarily due to high I^2^, publication bias, and serious doubts about directness.

**Table 4:** Grading of Recommendations, Assessment, Development, and Evaluation (GRADE) in included randomized controlled trials

Outcomes	Participants (studies)	Risk of bias	Inconsistency	Indirectness	Imprecision	Publication bias	Overall quality of evidence
Weight	1518 (27 studies)	no serious risk of bias	serious^1^	serious^2^	no serious imprecision	reporting bias strongly suspected^3^	⊕⊕⊝⊝ **LOW** ^1,2,3^ due to inconsistency, indirectness, publication bias, dose-response gradient
HDL	1352 (23 studies)	no serious risk of bias	no serious inconsistency^1^	no serious indirectness	no serious imprecision	undetected	⊕⊕⊕⊕ **HIGH**
LDL	1283 (22 studies)	no serious risk of bias	serious^1^	no serious indirectness	no serious imprecision	reporting bias strongly suspected^3^	⊕⊕⊕⊝ **LOW** ^1.3^ due to inconsistency
TG	549 (23 studies)	no serious risk of bias	serious^1^	no serious indirectness	no serious imprecision	undetected	⊕⊕⊕⊝ **MODERATE** ^1^ due to inconsistency
Fasting insulin	935 (20 studies)	no serious risk of bias	very serious^1^	no serious indirectness	no serious imprecision	undetected	⊕⊕⊝⊝ **LOW** ^1^ due to inconsistency
Fasting glucose	1086 (19 studies)	no serious risk of bias	serious^1^	no serious indirectness	no serious imprecision	undetected	⊕⊕⊕⊝ **MODERATE** ^1^ due to inconsistency

^1^ The effect sizes varied between studies, rated down by one level for high I^2^

^2^ There is a statistical difference between the direct comparison group and the final result (P<0.05)

^3^ Less eating time helps with weight loss

^4^ Inspection of funnel plot suggest publication bias

## Discussion

In this investigation, we assessed the potential benefits of TRE with varied eating windows. 27 RCTs were chosen for a systematic review, and a meta-analysis was subsequently performed. No distinct metabolic benefits were found associated with different eating windows. Therefore, our results indicate that diverse eating windows can elicit similar benefits on metabolic parameters. Contrary to this, prior research demonstrated varied advantageous outcomes with different TRE methods [18, 23–26], previous research indicates that fasting for longer than 12 h each day may have further cardiometabolic advantages [27]. Certain studies postulate that an overly condensed eating window dietary protocol might lead to diminished fat utilization [8], the metabolic transition, during which liver glycogen reserves are increasingly depleted and lipids/ketones are mobilized and oxidized, typically begins 12 h after the last meal. However, our study shows that therapies with various eating windows support comparable cardiometabolic outcomes in adults over 39 weeks. It may be related to the small number of studies with a fasting time of more than 10 h (2 studies). Given the diminished compliance resultant from an excessively restrictive eating window, we propose a suitable extension of the eating window.

The optimal temporal window for eating during TRE interventions is currently a subject of ongoing debate. TRE underscores the importance of daily circidian rhythms in regulating physiological responses in humans. Insulin sensitivity emerges as a key factor in the management of circadian rhythm, exhibiting a pattern of increase during daylight hours [28, 29]. The levels of fasting insulin serve as indicators of the condition of glucose metabolism. The presence of hyperinsulinemia is typically viewed as a sign of insulin resistance [30]. A thoughtful meta-analysis [25] has confirmed that in the general population, fasting insulin concentrations are independently linked to an increased risk of hypertension. Improvements in insulin sensitivity were seen in two trials that looked at early TRE windows; these studies used an early 6 h TRE window with dinner before 3 p.m. during five [31] and a 2-week, 8-hour TRE window from 8 a.m. to 4 p.m. [16]. Research examining late TRE windows, however, has yielded inconsistent results. In one study investigating the application of TRE within any 4-hour window between 4 PM and midnight over the course of 8 weeks, no noticeable variations in body composition alterations were observed. [32]. However, TRE windows of 4 h (eating from 3 to 7 p.m.) or 6 h (eating from 1 to 7 p.m.) were beneficial and over 8 weeks, significantly reduced body weight and insulin resistance. Owing to the variability of the TRE regimen, our study categorized daily eating windows but did not conduct subgroup analyses for differing durations within identical fasting windows of the day.

Our study suggests that confining daily caloric intake within a six-hour window might yield optimal results within the context of TRE. Its eating window maintains a degree of simplicity compared to the 16:8 and 14:10 methodologies, presenting fewer confounding elements. A notable aspect to examine is the interplay between internal chronobiological mechanisms and meal scheduling. This may elucidate why the effects of TRE appear time-dependent, given that food consumption is recognized as a “zeitgeber,“ or time-giver, known to synchronize peripheral clocks [33]. More specifically, the central clock, located in the suprachiasmatic nucleus, governs food intake and energy expenditure whereas tissue clocks (e.g., in the gut and liver) are involved in several processes, including glucose absorption and insulin regulation. Research to date suggests that limiting daily food intake to a six-to-eight-hour eating window may exhibit health benefits, including protection against certain types of cancers, heart disease, obesity, and hypertension [34]. The less-than-six-hour window group also demonstrated a drop in fasting glucose that might correlate with circadian rhythmicity in glucose tolerance [35–39].

The effects of TRE depend on the change in energy intake, participants in all TRE studies with a reduction in energy intake also demonstrated a reduction in body weight [40]. The study conducted by Mattson et al. showed no change in energy intake but decreased body weight in participants after TRE. However, this study restricted meal frequency to once a day, which is less frequent than other studies. Since meal frequency has been implicated in body weight management [41], this factor might account for the differing outcomes in Mattson et al.‘s study [42].

Although dietary changes have historically been thought of as helpful therapies for hypertriglyceridemia [43], the changes in blood lipid factors differed between TRE studies. The study found that TRE improved humans’ metabolic health, but there is no difference between different TRE schemes. The inclusion of fewer studies in the < 6 h group and = 12 h group, may have an impact on our research results.

## Conclusions

Our research examined data from TRE trials with various eating windows and suggested that the effects of various meal windows on enhancing metabolic health were similar, such as decreasing body weight and reducing fasting insulin. Therefore, our results suggest that different eating windows can promote similar benefits for metabolic parameters, such as blood lipids. Different eating windows appeared to have similar impacts on enhancing metabolic health, according to our research, which analyzed data from TRE studies with various eating windows. There appears to be no convincing evidence currently to suggest which eating windows are superior for the TRE. The evidence of main for our study is of moderate or low strength; therefore, these recommendations may change in the future if evidence of higher strength suggests the superiority of other treatments, further research should focus on promising interventions with inadequate strength of evidence and especially on the <6 h group and = 12 h group.

## Limitations

This study faces numerous limitations. First, despite the randomized nature of the trials, blinding participants to the intervention was not feasible. Second, the majority of trial participants were from a less healthy demographic because they may have already been interested in TRE or intended to improve their health through dietary modifications. Third, the number of participants was relatively low, possibly limiting their representativeness of the broader population. Fourth, potential TRE barriers went unanalyzed. Fifth, although TRE group participants were instructed to eat within a set period, the specific meal timings and duration largely varied, potentially influencing the outcomes. Thus, the impact of food consumption duration on TRE effects warrants further exploration. Sixth, alterations in eating periods within TRE groups might have modified fasting durations before to testing, thereby potentially affecting outcomes. Seventh, the small sample size was insufficiently powered to detect intergroup differences concerning certain secondary outcomes, necessitating a larger, corresponding clinical trial. Eighth, the scarcity of numerous comparative RCTs restricted this analysis, often making estimations predominantly or entirely dependent on indirect evidence. The small sample size and differences between direct and indirect estimates further compromised the original data. Ninth, participant baseline characteristics, study duration, meal frequency, and eating period length varied across the evaluated research. Such variations could yield different findings despite identical interventions. The challenges inherent to meta-analyses of observational research also pertain to this study. Despite statistical correction and NMA use, one cannot entirely exclude the presence of unmeasured confounders and potential treatment allocation bias. Consequently, considering the existing questions and gaps in TRE research literature, additional research is imperative.

### Electronic supplementary material

Below is the link to the electronic supplementary material.

Supplementary Material 1

Supplementary Material 2

## Data Availability

All data are incorporated into the article and its online supplementary material.

## References

[1] Seconda L, Baudry J, Alles B, Hamza O, Boizot-Szantai C, Soler LG, Galan P, Hercberg S, Lairon D, Kesse-Guyot E. Assessment of the Sustainability of the Mediterranean Diet Combined with Organic Food Consumption: An Individual Behaviour Approach, Nutrients, 9 (2017), 10.3390/nu9010061 10.3390/nu9010061 PMC5295105 28085096

[2] Carter S, Clifton PM, Keogh JB (2016). The effects of intermittent compared to continuous energy restriction on glycaemic control in type 2 Diabetes; a pragmatic pilot trial. Diabetes Res Clin Pract.

[3] Catenacci VA, Pan Z, Ostendorf D, Brannon S, Gozansky WS, Mattson MP, Martin B, MacLean PS, Melanson EL (2016). Troy Donahoo, a randomized pilot study comparing zero-calorie alternate-day fasting to daily caloric restriction in adults with obesity. Obes (Silver Spring).

[4] Harvie M, Wright C, Pegington M, McMullan D, Mitchell E, Martin B, Cutler RG, Evans G, Whiteside S, Maudsley S, Camandola S, Wang R, Carlson OD, Egan JM, Mattson MP, Howell A (2013). The effect of intermittent energy and carbohydrate restriction v. daily energy restriction on weight loss and metabolic Disease risk markers in overweight women. Br J Nutr.

[5] Harvie MN, Sims AH, Pegington M, Spence K, Mitchell A, Vaughan AA, Allwood JW, Xu Y, Rattray NJ, Goodacre R, Evans DG, Mitchell E, McMullen D, Clarke RB, Howell A (2016). Intermittent energy restriction induces changes in breast gene expression and systemic metabolism. Breast Cancer Res.

[6] Kirkham AA, Parr EB, Kleckner AS (2022). Cardiometabolic health impacts of time-restricted eating: implications for type 2 Diabetes, cancer and Cardiovascular Diseases. Curr Opin Clin Nutr Metab Care.

[7] Parvaresh A, Razavi R, Abbasi B, Yaghoobloo K, Hassanzadeh A, Mohammadifard N, Safavi SM, Hadi A, Clark CCT (2019). Modified alternate-day fasting vs. calorie restriction in the treatment of patients with metabolic syndrome: a randomized clinical trial. Complement Ther Med.

[8] Christensen RAG, Kirkham AA, Eating T-R. A novel and simple dietary intervention for primary and secondary Prevention of Breast Cancer and Cardiovascular Disease. Volume 13. Nutrients; 2021. 10.3390/nu13103476 10.3390/nu13103476 PMC8537890 34684476

[9] Liu L, Chen W, Wu D, Hu F (2022). Metabolic efficacy of time-restricted eating in adults: a systematic review and Meta-analysis of Randomized controlled trials. J Clin Endocrinol Metab.

[10] Moon S, Kang J, Kim SH, Chung HS, Kim YJ, Yu JM, Cho ST, Oh CM, Kim T. Beneficial effects of Time-restricted eating on metabolic Diseases: a systemic review and Meta-analysis. Nutrients. 2020;12. 10.3390/nu12051267 10.3390/nu12051267 PMC7284632 32365676

[11] Queiroz JDN, Macedo RCO, Dos Santos GC, Munhoz SV, Machado CLF, de Menezes RL, Menzem EN, Moritz CEJ, Pinto RS, Tinsley GM, de Oliveira AR. Cardiometabolic effects of early v. delayed time-restricted eating plus energetic restriction in adults with overweight and obesity: an exploratory randomised clinical trial. Br J Nutr. 2022;1–13. 10.1017/S0007114522001581 10.1017/S0007114522001581 35614845

[12] Carlson O, Martin B, Stote KS, Golden E, Maudsley S, Najjar SS, Ferrucci L, Ingram DK, Longo DL, Rumpler WV, Baer DJ, Egan J, Mattson MP. Impact of reduced meal frequency without caloric restriction on glucose regulation in healthy, normal-weight middle-aged men and women, metabolism: clinical and experimental, (2007) 1729–34. 10.1016/j.metabol.2007.07.018 PMC2121099 17998028

[13] Gill S, Panda S. A Smartphone App reveals erratic diurnal eating patterns in humans that can be modulated for Health benefits, Cell Metabol, (2015) 789–98. 10.1016/j.cmet.2015.09.005 PMC4635036 26411343

[14] Moro T, Tinsley G, Longo G, Grigoletto D, Bianco A, Ferraris C, Guglielmetti M, Veneto A, Tagliabue A, Marcolin G, Paoli A (2020). Time-restricted eating effects on performance, immune function, and body composition in elite cyclists: a randomized controlled trial. J Int Soc Sports Nutr.

[15] Stote KS, Baer DJ, Spears K, Paul DR, Harris GK, Rumpler WV, Strycula P, Najjar SS, Ferrucci L, Ingram DK, Longo DL, Mattson MP. A controlled trial of reduced meal frequency without caloric restriction in healthy, normal-weight, middle-aged adults, the American journal of clinical nutrition, (2007) 981–8. 10.1093/ajcn/85.4.981 PMC2645638 17413096

[16] Tinsley GM, Moore ML, Graybeal AJ, Paoli A, Kim Y, Gonzales JU, Harry JR, VanDusseldorp TA, Kennedy DN, Cruz MR (2019). Time-restricted feeding plus resistance training in active females: a randomized trial. Am J Clin Nutr.

[17] Che T, Yan C, Tian D, Zhang X, Liu X, Wu Z (2021). Time-restricted feeding improves blood glucose and insulin sensitivity in overweight patients with type 2 Diabetes: a randomised controlled trial. Nutr Metab (Lond).

[18] Cienfuegos S, Gabel K, Kalam F, Ezpeleta M, Wiseman E, Pavlou V, Lin S, Oliveira ML, Varady KA (2020). Effects of 4- and 6-h time-restricted feeding on Weight and Cardiometabolic Health: a randomized controlled trial in adults with obesity. Cell Metab.

[19] Moher D, Liberati A, Tetzlaff J, Altman DG, Group P (2009). Preferred reporting items for systematic reviews and meta-analyses: the PRISMA Statement. Open Med.

[20] Page MJ, Moher D, McKenzie JE (2022). Introduction to preferred reporting items for systematic reviews and meta-analyses 2020 and implications for research synthesis methodologists. Res Synth Methods.

[21] R. G, Network meta-analysis, electrical networks and graph theory, Res Synthesis Methods, (2012) 312–24. 10.1002/jrsm.1058 26053424

[22] Puhan MA, Schunemann HJ, Murad MH, Li T, Brignardello-Petersen R, Singh JA, Kessels AG, Guyatt GH, Group GW. A GRADE Working Group approach for rating the quality of treatment effect estimates from network meta-analysis, BMJ, 349 (2014) g5630, 10.1136/bmj.g5630 10.1136/bmj.g5630 25252733

[23] Anton SD, 2, Lee SA, Donahoo WT, McLaren C, Manini T, Leeuwenburgh C, 2, Pahor M. The Effects of Time Restricted Feeding on Overweight, Older Adults: A Pilot Study, Nutrients, (2019) 1500. 10.3390/nu11071500 PMC6682944 31262054

[24] Chow LS, Manoogian ENC, Alvear A, Fleischer JG, Thor H, Dietsche K, Wang Q, Hodges JS, Esch N, Malaeb S, Harindhanavudhi T, Nair KS, Panda S, Mashek DG. Time-Restricted Eating effects on body composition and metabolic measures in humans who are overweight: a feasibility study, obesity (Silver Spring), 28 (2020) 860–9, 10.1002/oby.22756 10.1002/oby.22756 PMC7180107 32270927

[25] Sutton EF, Beyl R, Early KS, Cefalu WT, Ravussin E, Peterson CM (2018). Early Time-restricted feeding improves insulin sensitivity, blood pressure, and oxidative stress even without weight loss in men with Prediabetes. Cell Metab.

[26] Wilkinson13 MJ, Manoogian ENC23, Zadourian1 A, Lo1 H, Fakhouri2 S, Shoghi2 A, Wang2 X, Fleischer2 JG, Navlakha S2, Panda24 S. P.R. Taub1, Ten-Hour Time-Restricted Eating Reduces Weight, Blood Pressure, and Atherogenic Lipids in Patients with Metabolic Syndrome, Cell Metabolism, (2020) 92–104(e105). 10.1016/j.cmet.2019.11.004 PMC6953486 31813824

[27] Patterson RE, Sears DD (2017). Metabolic effects of Intermittent Fasting. Annu Rev Nutr.

[28] Paoli A, Tinsley G, Bianco A, Moro T. The Influence of Meal Frequency and Timing on Health in Humans: The Role of Fasting, Nutrients, 11 (2019), 10.3390/nu11040719 10.3390/nu11040719 PMC6520689 30925707

[29] W. K.L., The metabolic syndrome: evolving evidence that thiazolidinediones provide rational therapy, Diabetes Obes Metabolism, (2006) 365–80. 10.1111/j.1463-1326.2005.00522.x 16776743

[30] Wang F, Han L, Hu D (2017). Fasting insulin, insulin resistance and risk of Hypertension in the general population: a meta-analysis. Clin Chim Acta.

[31] Jones R, Pabla P, Mallinson J, Nixon A, Taylor T, Bennett A, Tsintzas K (2020). Two weeks of early time-restricted feeding (eTRF) improves skeletal muscle insulin and anabolic sensitivity in healthy men. Am J Clin Nutr.

[32] Berglund L, Brunzell JD, Goldberg AC, Goldberg IJ, Sacks F, Murad MH, Stalenhoef AF, s., Endocrine. Evaluation and treatment of hypertriglyceridemia: an Endocrine Society clinical practice guideline, J Clin Endocrinol Metab, 97 (2012) 2969–2989, 10.1210/jc.2011-3213 10.1210/jc.2011-3213 PMC3431581 22962670

[33] Flanagan A, Bechtold DA, Pot GK, Johnston JD (2021). Chrono-nutrition: from molecular and neuronal mechanisms to human epidemiology and timed feeding patterns. J Neurochem.

[34] Ruddick-Collins LC, Morgan PJ, Johnstone AM (2020). Mealtime: a circadian disruptor and determinant of energy balance?. J Neuroendocrinol.

[35] Poggiogalle E, Jamshed H, Peterson CM (2018). Circadian regulation of glucose, lipid, and energy metabolism in humans. Metabolism.

[36] Palomar-Cros A, Srour B, Andreeva VA, Fezeu LK, Bellicha A, Kesse-Guyot E, Hercberg S, Romaguera D, Kogevinas M, Touvier M (2023). Associations of meal timing, number of eating occasions and night-time fasting duration with incidence of type 2 Diabetes in the NutriNet-Sante cohort. Int J Epidemiol.

[37] A GRADE Working Group approach for (2015). Rating the quality of treatment effect estimates from network meta-analysis. BMJ.

[38] Xu S, Qiu Y, Tao J (2021). The challenges and optimization of cell-based therapy for Cardiovascular Disease. J Transl Int Med.

[39] Chen M, Chen W (2022). Single-nucleotide polymorphisms in Medical Nutritional Weight loss: challenges and future directions. J Transl Int Med.

[40] Xie SY, Ye Z. Y, Randomized controlled trial for time-restricted eating in healthy volunteers without obesity, Nat Commun, (2022). 10.1038/s41467-022-28662-5 PMC8864028 35194047

[41] Liu HY, Huang D, Yang C, Wei S, Zhang X, Guo P, Lin D, Xu J, Li B, He C, He H, Liu J, Shi S, Xue L, Zhang Y (2022). Calorie restriction with or without time-restricted eating in weight loss. N Engl J Med.

[42] Kulovitz MG, Kravitz LR, Mermier C, Gibson AL, Conn CA, Kolkmeyer D, Kerksick CM. Potential role of meal frequency as a strategy for weight loss and health in overweight or obese adults, Nutrition, (2014) 386–92. 10.1016/j.nut.2013.08.009 24268866

[43] Kord-Varkaneh H, Salehi-Sahlabadi A, Tinsley GM, Santos HO, Hekmatdoost A (2023). Effects of time-restricted feeding (16/8) combined with a low-sugar diet on the management of non-alcoholic fatty Liver Disease: a randomized controlled trial. Nutrition.

[44] Moro T, Tinsley G, Pacelli FQ, Marcolin G, Bianco A, Paoli A (2021). Twelve months of Time-restricted eating and resistance training improves inflammatory markers and cardiometabolic risk factors. Med Sci Sports Exerc.

[45] Kotarsky CJ, Johnson NR, Mahoney SJ, Mitchell SL, Schimek RL, Stastny SN, Hackney KJ (2021). Time-restricted eating and concurrent exercise training reduces fat mass and increases lean mass in overweight and obese adults. Physiol Rep.

[46] He M, Wang J, Liang Q, Li M, Guo H, Wang Y, Deji C, Sui J, Wang YW, Liu Y, Zheng Y, Qian B, Chen H, Ma M, Su S, Geng H, Zhou WX, Guo X, Zhu WZ, Zhang M, Chen Z, Rensen PCN, Hui CC, Wang Y, Shi B (2022). Time-restricted eating with or without low-carbohydrate diet reduces visceral fat and improves metabolic syndrome: a randomized trial. Cell Rep Med.

[47] Brady AJ, Langton HM, Mulligan M, Egan B (2021). Effects of 8 wk of 16:8 time-restricted eating in male Middle- and Long-Distance runners. Med Sci Sports Exerc.

[48] Lin YJ, Wang YT, Chan LC, Chu NF (2022). Effect of time-restricted feeding on body composition and cardio-metabolic risk in middle-aged women in Taiwan. Nutrition.

[49] Cai H, Qin YL, Shi ZY, Chen JH, Zeng MJ, Zhou W, Chen RQ, Chen ZY (2019). Effects of alternate-day fasting on body weight and dyslipidaemia in patients with non-alcoholic fatty Liver Disease: a randomised controlled trial. BMC Gastroenterol.

[50] Lowe DA, Wu N, Rohdin-Bibby L, Moore AH, Kelly N, Liu YE, Philip E, Vittinghoff E, Heymsfield SB, Olgin JE, Shepherd JA, Weiss EJ (2020). Effects of Time-restricted eating on weight loss and other metabolic parameters in women and men with overweight and obesity: the TREAT Randomized Clinical Trial. JAMA Intern Med.

[51] Jamshed H, Steger FL, Bryan DR, Richman JS, Warriner AH, Hanick CJ, Martin CK, Salvy SJ, Peterson CM (2022). Effectiveness of early time-restricted eating for weight loss, Fat loss, and Cardiometabolic Health in adults with obesity: a Randomized Clinical Trial. JAMA Intern Med.

[52] Lao BN, Luo JH, Xu XY, Fu LZ, Tang F, Ouyang WW, Xu XZ, Wei MT, Xiao BJ, Chen LY, Wu YF, Liu XS (2023). Time-restricted feeding’s effect on overweight and obese patients with chronic Kidney Disease stages 3–4: a prospective non-randomized control pilot study. Front Endocrinol (Lausanne).

[53] Haganes KL, Silva CP, Eyjolfsdottir SK, Steen S, Grindberg M, Lydersen S, Hawley JA, Moholdt T (2022). Time-restricted eating and exercise training improve HbA1c and body composition in women with overweight/obesity: a randomized controlled trial. Cell Metab.

[54] Manoogian ENC, Zadourian A, Lo HC, Gutierrez NR, Shoghi A, Rosander A, Pazargadi A, Ormiston CK, Wang X, Sui J, Hou Z, Fleischer JG, Golshan S, Taub PR, Panda S (2022). Feasibility of time-restricted eating and impacts on cardiometabolic health in 24-h shift workers: the Healthy heroes randomized control trial. Cell Metab.

[55] Andriessen C, Fealy CE, Veelen A, van Beek SMM, Roumans KHM, Connell NJ, Mevenkamp J, Moonen-Kornips E, Havekes B, Schrauwen-Hinderling VB, Hoeks J, Schrauwen P (2022). Three weeks of time-restricted eating improves glucose homeostasis in adults with type 2 Diabetes but does not improve insulin sensitivity: a randomised crossover trial. Diabetologia.

[56] Thomas EA, Zaman A, Sloggett KJ, Steinke S, Grau L, Catenacci VA, Cornier MA, Rynders CA (2022). Early time-restricted eating compared with daily caloric restriction: a randomized trial in adults with obesity. Obes (Silver Spring).

[57] Phillips NE, Mareschal J, Schwab N, Manoogian ENC, Borloz S, Ostinelli G, Gauthier-Jaques A, Umwali S, Gonzalez Rodriguez E, Aeberli D, Hans D, Panda S, Rodondi N, Naef F, Collet TH. The Effects of Time-Restricted Eating versus Standard Dietary Advice on Weight, Metabolic Health and the Consumption of Processed Food: A Pragmatic Randomised Controlled Trial in Community-Based Adults, Nutrients, 13 (2021), 10.3390/nu13031042 10.3390/nu13031042 PMC8004978 33807102

[58] Rona A, Robertson TM, Denise RM, Johnston JD (2018). A pilot feasibility study exploring the effects of a moderate time-restricted feeding intervention on energy intake, adiposity and metabolic physiology in free-living humans. J Nutritional Sci.

[59] Mayra ST, Chondropoulos K, De Leon A, Kravat N, Johnston CS (2022). The feasibility and preliminary efficacy of early time-restricted eating on diet quality in college students: a randomized study. Obes Res Clin Pract.

