# Supplementary material for: Effects of time-restricted eating with different eating windows on human metabolic health: pooled analysis of existing cohorts
Source: Diabetol Metab Syndr. 2023 Oct 24;15:209. doi: 10.1186/s13098-023-01190-y (PMC10594936; doi:10.1186/s13098-023-01190-y)
Supplement: Supplementary file 2 — Supplementary Material 2 [file 13098_2023_1190_MOESM2_ESM.docx]

| Database | Search term (inception to November 6, 2022) | Number of Studies |
| --- | --- | --- |
| PubMed Title and abstract | (restricted eating[Title/Abstract] OR Intermittent Fasting[Title/Abstract] OR Fasting-Mimicking Diets[Title/Abstract] OR time-restricted eating[Title/Abstract] OR alternate day fasting[Title/Abstract] OR intermittent fasting[Title/Abstract] OR fasting[Title/Abstract] OR time restricted feeding[Title/Abstract] OR meal skipping[Title/Abstract] OR reduced meal frequency[Title/Abstract]) | 1846 |
| EMBASE | (restricted eating[Title/Abstract] OR Intermittent Fasting[Title/Abstract] OR Fasting-Mimicking Diets[Title/Abstract] OR time-restricted eating[Title/Abstract] OR alternate day fasting[Title/Abstract] OR intermittent fasting[Title/Abstract] OR fasting[Title/Abstract] OR time restricted feeding[Title/Abstract] OR meal skipping[Title/Abstract] OR reduced meal frequency[Title/Abstract]) | 765 |
| Cochrane Library | (restricted eating[Title/Abstract] OR Intermittent Fasting[Title/Abstract] OR Fasting-Mimicking Diets[Title/Abstract] OR time-restricted eating[Title/Abstract] OR alternate day fasting[Title/Abstract] OR intermittent fasting[Title/Abstract] OR fasting[Title/Abstract] OR time restricted feeding[Title/Abstract] OR meal skipping[Title/Abstract] OR reduced meal frequency[Title/Abstract]) | 448 |

**Table S1**. Electronic search strategy.

**Table S2**. Individual study data (pre-post intervention) for all outcome measures included in NMA

|  |  | **Pre-Post Outcomes Measures: mean (SD)** | | | | | | | | | | | | | | | | | |
| --- | --- | --- | --- | --- | --- | --- | --- | --- | --- | --- | --- | --- | --- | --- | --- | --- | --- | --- | --- |
|  |  | **Body weight** | | | **TG** | | | **LDL** | | | **HDL** | | | **Fasting glucose** | | | **Fasting insulin** | | |
| **Author** | **Exercise category** | **pre** | **post** | **Mean differe nce (SD)** | **pre** | **post** | **Mean differe nce (SD)** | **pre** | **post** | **Mean differe nce (SD)** | **pre** | **post** | **Mean differe nce (SD)** | **pre** | **post** | **Mean differe nce (SD)** | **pre** | **post** | **Mean difference ±SD** |
| Kord-Varkaneh H et al.(2022) | TRE | 83.75±12.71 | 80.54±12.12 |  | 201.50±35.34 | 133.27±48.67 |  | 104.63±27.26 | 84.04±26.27 |  | 38.18±5.65 | 39.81±5.72 |  |  |  |  | 9.16±3.22 | 11.44±4.65 |  |
|  | CON | 89.33± 18.47 | 88.31±11.01 |  | 187.6±73.61 | 180.72±49.49 |  | 93.73±31.77 | 97.45±35.46 |  | 34.82±7.65 | 33.73±6.70 |  |  |  |  | 10.78±3.78 | 12.15±5.25 |  |
| Moro T et al. (2021) | TRE | 83.22±5.92 | 82.89±6.04 |  | 118.30±15.08 | 114.00±9.88 |  | 118.30±15.08 | 111.80±11.53 |  | 53.70±2.67 | 57.40±2.46 |  | 95.10±5.30 | 86.20±5.47 |  | 2.59±0.41 | 1.87±0.55 |  |
|  | CON | 84.64±5.76 | 85.00±5.40 |  | 119.90±11.01 | 118.00±11.37 |  | 119.90±11.01 | 117.50±11.26 |  | 53.60±2.07 | 55.10±2.23 |  | 95.50±4.33 | 95.60±4.65 |  | 2.48±0.31 | 2.39±0.45 |  |
| Kotarsky C J et al.(2021) | TRE | 83±3 | 79±3 |  |  |  |  |  |  |  | 55±3 | 54±3 |  |  |  |  | 11±1 | 9±2 |  |
|  | CON | 82±3 | 83±3 |  |  |  |  |  |  |  | 55±6 | 55±4 |  |  |  |  | 13±2 | 10±1 |  |
| He M et al.(2022) | TRE | 84.3±2.2 | 81.2±2.2 |  | 152.39*104.54 | 143.53*109.86 |  | 116.4*4.64 | 121.52*5.42 |  | 42.52±1.16 | 43.68±1.16 |  | 90.9*16.02 | 85.68*18.18 |  | 26.99*18.87 | 26.5*9.43 |  |
|  | CON | 84.7±2.0 | 82.3±2.4 |  | 139.98*101.89 | 92.14*62.90 |  | 116.1*5.03 | 126.55*5.42 |  | 43.68±1.16 | 44.84±1.16 |  | 91.8*17.46 | 93.96*19.98 |  | 25.59*18.8 | 23.7*6.54 |  |
| Brady A J et al. (2021) | TRE | 72.17±6.68 | 71.44±0.48 |  | 79.7±48.1 | 81.0±13.5 |  |  |  |  |  |  |  | 93.96±7.56 | 94.32±2.34 |  | 3.55±0.73 | 3.44±0.22 |  |
|  | CON | 73.13±6.06 | 73.36±0.57 |  | 76.1±37.3 | 74.2±16.1 |  |  |  |  |  |  |  | 89.46±6.48 | 90.54±2.7 |  | 3.44±0.60 | 3.11±0.26 |  |
| Queiroz J.D.N et al. (2022) | TRE | 83·5*24.86 | 79.3*23.95 |  | 100*62.54 | 100*62.54 |  | 114*63.57 | 106*58.53 |  | 44*15.92 | 41*11.92 |  | 97*21.43 | 93*23.4 |  | 8*6.01 | 6*4.69 |  |
|  | CON | 81·6*27.19 | 77.68*24.67 |  | 110*61.62 | 110*61.62 |  | 119*65.43 | 105*46.4 |  | 53*23.78 | 45*19.74 |  | 95*24.74 | 92*13.01 |  | 10*5.38 | 7*2.2 |  |
| Chow L S et al. (2020) | TRE | 95.2±22.6 | 91.6*21.5 |  | 144*54 | 106*39 |  | 95*24 | 104*31 |  | 50*14 | 51*14 |  | 95*10 | 87*9 |  | 11*6 | 11*7 |  |
|  | CON | 100.9±28.1 | 99.4*28.1 |  | 87*21 | 87*27 |  | 105*19 | 106*19 |  | 60*18 | 67*19 |  | 95*13 | 88*3 |  | 10*5 | 10*8 |  |
| Lin Y J et al. (2021) | TRE | 65.9±9.7 | 63.2± 9.8 |  | 104.1±38.8 | 96.5±41.1 |  | 108.3±32.5/ | 106.8±27.3 |  | 63.9±14.2 | 65.4±13.8 |  | 88.3±7.6 | 92.6±9.1 |  | 7.9±3.3 | 8.9±4.4 |  |
|  | CON | 65.8±8.8 | 64.2 ± 8.5 |  | 107.9±47.3 | 97.1± 39.7 |  | 107.8±26.4 | 111.6±29.0 |  | 63.2±11.8 | 63.0±9.6 |  | 89.5±8.6 | 91.3±10.1 |  | 11.1±5.5 | 10.7±7.0 |  |
| Moro T et al.(2020) | TRE | 67.04±5.03 | 65.78±4.93 |  | 69.75±26.13 | 54.13±20.23 |  |  |  |  |  |  |  | 94.63±5.45 |  |  | 5.25±1.75 | 3.88±1.96 |  |
|  | CON | 72.27±6.24 | 72.50±6.45 |  | 70.50±54.51 | 70.38±56.89 |  |  |  |  |  |  |  | 91.00±5.15 |  |  | 5.63±1.60 | 5.50±2.88 |  |
| Cai H et al.(2019) | TRE | 74.98 ± 8.02 | 71.31±7.04 |  | 256.86±155 | 201.06±155 |  | 105.56±34.02 | 45.63±18.17 |  | 44.85±17.40 | 93.78±19.08 |  | 92.16±14.76 |  |  |  |  |  |
|  | CON | 72.94±8.00 | 70.32±6.75 |  | 234.72±149.68 | 201.94±150.57 |  | 98.60±30.54 | 49.8±21.26 |  | 44.85±19.33 | 93.24±1.29 |  | 91.62±16.2 |  |  |  |  |  |
| Tinsley G M et al. (2019) | TRE | 63.8±8.5 |  |  | 88±10 | 93±12 |  | 97±7 | 89±7 |  | 64±4 | 65±5 |  | 89±3 | 89±3 |  | 12±3 |  |  |
|  | CON | 64.6±8.8 |  |  | 83±9 | 75±13 |  | 92±7 | 99±8 |  | 69±4 | 69±6 |  | 93±3 | 91±4 |  | 13±4 |  |  |
| Lowe D A et al.(2020) | TRE | 99.3±16.9 | 90.9*10.21 |  | 127.2*25 | 116.9*61.5 |  | 122.1*11.5 | 122.7*26.9 |  | 54.7*5.4 | 54.0*12.9 |  | 91.7*3.1 | 90.6*9.09 |  | 14.7*3.3 | 11.9*8.13 |  |
|  | CON | 99.1±15.1 | 92.4*8.27 |  | 133.4*25 | 136.0*63.48 |  | 126.4*11.4 | 124.2*27.6 |  | 50.1*5.4 | 50.7*13.37 |  | 93.9*3.4 | 94.2*4.12 |  | 12.4*3.3 | 14.8*8.37 |  |
| Jamshed H et al. (2022) | TRE | 112.3±20.1 |  | -6.3*3.08 | 117±67 |  | -3*61.6 | 119*29 |  | -5*27.38 | 62*14 |  | −4*6.84 | 107*17 |  | -8*13.69 | 22.0*17.2 |  | -6.4*15.06 |
|  | CON | 105.3±20.7 |  | -4*3.76 | 118±62 |  | -9*61.6 | 117*26 |  | −9*27.38 | 61*16 |  | -4*13.69 | 103*14 |  | -6*13.69 | 17.2*9.5 |  | -1.8*6.16 |
| Liu D et al. (2022) | TRE |  |  | −9.4*6.19 |  |  | -44.8*66.16 | 130.7±29.9 |  | −5.9*25.39 | 46.1±10.2 |  | 4.2 *7.89 | 90.9±11.0 |  | −15.4*35.42 |  |  |  |
|  | CON |  |  | −8.9*6.19 |  |  | −31.7*64.03 | 129.6±33.7 |  | −11.3*24.54 | 45.1±12.7 |  | 2.7*7.47 | 92.1±17.1 |  | −10.6*34.14 |  |  |  |
| Xie Z et al.(2022) | TRE | 61.1±8.8 |  | -1.6±1.4 |  |  | 0.02±0.48 |  |  | 0.00±18.94 |  |  | -1.16±7.73 |  |  |  |  |  |  |
|  | CON | 61.2±9.9 |  | 0.3±1.2 |  |  | 0.07±0.38 |  |  | -3.09±13.91 |  |  | 1.93±6.57 |  |  |  |  |  |  |
| Haganes K L et al.(2022) | TRE | 91.0±10.8 | 89.4*12.3 |  |  | 106.32*44.3 |  |  | 127.71*27.09 |  | 50.31*11.61 | 50.31*7.74 |  | 90*9 | 88.2*7.2 |  |  | 4.9*0.5 |  |
|  | CON | 95.0±11.2 | 94.2*12.0 |  |  | 97.46*44.3 |  |  | 127.71*42.57 |  | 50.31*11.61 | 50.31*11.61 |  | 90*7.2 | 90*7.2 |  |  | 5*0.4 |  |
| Manoogian E et al.(2022) | TRE | 107.1±5.35 | 86.31*13.78 |  | 94.24*3.3 | 92.53*65.09 |  | 122.36*7.1 | 123.12*29.15 |  | 59.53*4.4 | 59.66 *17.09 |  | 92.31*1.9 | 92.5*6.16 |  | 4.90*0.75 | 4.84*2.43 |  |
|  | CON | 108.07±1.75 | 88.86*14.88 |  | 86.67*5.7 | 83.5*49.74 |  | 121.47*7.7 | 118.86*33.34 |  | 55.48*2.5 | 56.76*12.12 |  | 92.47*1.8 | 92.61*7.35 |  | 4.71*0.71 | 4.85*0.56 |  |
| Che T et al.(2021) | TRE | 75.06±4.42 |  |  | 491.58±97.43 |  |  | 141.91±47.9 |  |  | 47.95±11.98 |  |  |  |  |  | 8.83±1.19 |  |  |
|  | CON | 74.68±4.35 |  |  | 471.21±97.43 |  |  | 138.05±40.21 |  |  | 46.01±13.14 |  |  |  |  |  | 8.84±1.17 |  |  |
| Andriessen C et al.(2022) | TRE |  |  |  |  | 85.07±7.73 |  |  |  |  |  |  |  | 142.20±23.4 | 144±5.4 |  |  | 15.48±2.89 |  |
|  | CON |  |  |  |  | 81.2±11.60 |  |  |  |  |  |  |  | 142.20±23.4 | 160.2±9 |  |  | 13.54±1.93/ |  |
| Thomas E A et al.(2021 | TRE | 96.1±18.1 | 91.2*18.6 |  |  |  | -18.98*34.54 |  |  | -0.88*16.98 |  |  | -0.41*9.17/ |  |  |  |  |  |  |
|  | CON | 93.4±18.4 | 88.9*19.7 |  |  |  | 8.74*44.59 |  |  | -7.54*24.59 |  |  | -0.15*6.57 |  |  |  |  |  |  |
| Phillips N.E et al.(2021) | TRE | 79.6±15.9 | 78.0*16.4 |  | 119.61*58.47 | 109.86*50.50 |  |  |  |  | 56.84*14.30 |  |  | 93.78*10.08 |  |  |  |  |  |
|  | CON | 77.5±13.8 | 76.4*12.7 |  | 109.86*50.50 | 91.25*31.89 |  |  |  |  | 55.68* 11.21 |  |  | 93.06*12.06 |  |  |  |  |  |
| de Oliveira M P I et al.(2021) | TRE | 81.25±13.51 | 79.73*4.1 |  |  |  |  |  |  |  |  |  |  |  |  |  |  |  |  |
|  | CON | 80.25±9.40 | 80.67*5.4 |  |  |  |  |  |  |  |  |  |  |  |  |  |  |  |  |
| Rona A et al.(2018) | TRE | 86.2±5.2 | 85.5*5.2 |  | 95.68*6.20 | 112.52*19.49 |  | 138.01*19.33 | 120.61*11.98 |  | 41.753*3.86 | 48.71*23.58 |  | 87.3*1.44 | 86.22*0.54 |  | 5.08*1.6 | 6.83*1.5 |  |
|  | CON | 77.8±7.6 | 77.3*7.7 |  | 79.74*12.40 | 103.66*9.74 |  | 117.91*14.304 | 131.444*15.85 |  | 40.98*4.63 | 44.84*6.18 |  | 83.16*2.16 | 88.56*2.88 |  | 4.71*1.88 | 6.89*1.4 |  |
| Cienfuegos S et al.(2020) | TRE | 100±4 |  | -3.2±0.4 | 92 ± 10 |  | -1.9±6.7 | 96 ± 6 |  | -2.6±5.7 | 57 ± 4 |  | -2.4±1.3 | 88 ± 2 |  | −5.0±3.8 | 13 ± 2 |  | -2.3±1.5 |
|  | TRE | 99±4 |  | -3.2±0.4 | 94 ± 7 |  | -1.9±6.7 | 107 ± 8 |  | -4.8±5.1 | 54 ± 3 |  | -0.8±1.4 | 93 ± 2 |  | 2.3±2.0 | 18 ± 3 |  | -1.9±1.1 |
|  | CON | 94±3 |  | 0.1±0.4 | 87 ± 10/ |  | 4.5±3.2 | 104 ± 6 |  | 2.0±3.7 | 58 ± 4/ |  | −0.7±1.0 | 96 ± 3 |  | 2.6±2.6 | 13 ± 2 |  | 3.5±1.4 |
| Mayra S T et al.(2022) | TRE | 68.3±11.3 | 58.4 ± 6.4 |  | 61.2±19.1 | 61.3±26.5 |  | 101.3± 27.4 | 93.8 ± 19.8 |  | 63.7±14.7 | 61.1 ± 10.5 |  | 95.3 ± 7.0 | 90.6±5.7 |  | 11.9 ± 3.5 | 8.9 ± 2.5 |  |
|  | CON | 67.6±10.6 | 68.3 ±11.3 |  | 69.0±25.8 | 74.1±42.1 |  | 108.9± 20.8 | 108.9 ± 23.1 |  | 60.0 ± 9.7 | 61.1 ± 12.3 |  | 97.7 ± 6.5 | 99.0±5.8 |  | 8.7 ± 4.1 | 12.3 ± 0.0 |  |
| Sutton E F et al. (2018) | TRE |  |  | -1.4±1.3 |  |  | -44.46*70.71 |  |  | -2.5*2.82 |  |  | -7.07*19.8 |  |  | -8.08*12.7 |  |  | 38.69*58.2 |
|  | CON |  |  | 0.5±0.3 |  |  | -14.82*14.99 |  |  | -1.9*2.82 |  |  | -8.93*14.14 |  |  | -13.04*12.7 |  |  | -12.69*26.45 |

**Table S3.** Individual study data (pre-post intervention) for all outcome measures not included in NMA

| **Pre-Post Outcomes Measures: mean (SD)** | | | | | | | | | | | | | | | | | | | |
| --- | --- | --- | --- | --- | --- | --- | --- | --- | --- | --- | --- | --- | --- | --- | --- | --- | --- | --- | --- |
|  |  | **HOMA2-IR** | | | **SBP** | | | **DBP** | | | **BMI (kg/m 2 )** | | | **Hb1Ac (%)** | | | **TC** | | |
| **Author** | **Exercise category** | **pre** | **post** | **Mean differe nce (SD)** | **pre** | **post** | **Mean differe nce (SD)** | **pre** | **post** | **Mean differe nce (SD)** | **pre** | **post** | **Mean differe nce (SD)** | **pre** | **post** | **Mean differe nce (SD)** | **pre** | **post** | **Mean difference ±SD** |
| Moro T et al. (2021) | TRE | 0.61±0.10 | 0.40±0.13 |  |  |  |  |  |  |  | 29.8±0.8 | 28.8±0.8 |  |  |  |  | 194.90±8.44 | 192.00±11.08 |  |
|  | CON | 0.58±0.08 | 0.57±0.12 |  |  |  |  |  |  |  | 29.4±0.8 | 29.3±0.9 |  |  |  |  | 198.30±13.28 | 197.00±11.29 |  |
| Kotarsky C J et al.(2021) | TRE |  |  |  | 122±3 | 116±3 |  | 81 ± 2 | 80±2 |  |  |  |  | 4.6±0.2 | 4.6±0.3 |  |  |  |  |
|  | CON |  |  |  | 120±2 | 118±3 |  | 83 ± 1 | 79±2 |  |  |  |  | 4.7±0.2 | 4.3±0.2 |  |  |  |  |
| He M et al.(2022) | TRE |  | 5.41*1.35 |  | 136 ± 2 | 137±2 |  | 87 ± 2 | 85±2 |  | 29.6±5 | 28.1±0.4 |  | 5.6*0.6 | 5.6*0.6 |  | 184.06±5.02 | 185.22±5.41 |  |
|  | CON |  | 4.64*0.09 |  | 130±2 | 130±3 |  | 82 ± 2 | 81±2 |  | 29.3±0.5 | 28.3±0.4 |  | 5.7*0.6 | 5.7*0.6 |  | 182.52±5.4 | 189.86±5.8 |  |
| Brady A J et al. (2021) | TRE | 0.81 ± 0.23 | 0.81±0.07 |  |  |  |  |  |  |  |  |  |  |  |  |  |  |  |  |
|  | CON | 0.69 ± 0.14 | 0.70±0.08 |  |  |  |  |  |  |  |  |  |  |  |  |  |  |  |  |
| Queiroz J.D.N et al. (2022) | TRE | 8*2.5 | 6*3.68 |  |  | 110*8 |  | 76*8 |  |  | 30.8*3 |  |  |  |  |  | 179*70.34 |  | 167*67.26 |
|  | CON | 10*2 | 7*0.92 |  |  | 107*11 |  | 75*8 |  |  | 30.1*3 |  |  |  |  |  | 197*82.58 |  | 172*33.11 |
| Chow L S et al. (2020) | TRE | 2.5*1.6 | 2.4*1.9 |  | 132*13.0 | 121*16 |  | 85*4 | 79*15 |  | 33.8*7.6 |  |  |  | 5.4*0.4 |  |  |  |  |
|  | CON | 2.3*1.2 | 2.2*1.9 |  | 123 *3 | 115*12 |  | 79*8 | 72*7 |  | 34.4*7.8 |  |  |  | 5.6*0.4 |  |  |  |  |
| Lin Y J et al. (2021) | TRE |  | 2.1±1.0 |  | 121.2± 16.1 | 118.1±11.8 |  | 75.3±11.2 | 70.5±9.4 |  | 25.9±3.7 | 24.5±3.3 |  |  |  |  | 186.6±41.4 | 189.7±31.9 |  |
|  | CON |  | 2.6±2.1 |  | 121.1±12.9 | 121.2± 25.6 |  | 71.2±10.1 | 74.6±13.7 |  | 25.7±3.8 | 25.2±3.6 |  |  |  |  | 188.3±27.9 | 188.4±29.3 |  |
| Moro T et al.(2020) | TRE |  |  |  |  |  |  |  |  |  | 21.85 ± 1.65 |  |  |  |  |  | 171.00±18.52 | 181.38± 41.59 |  |
|  | CON |  |  |  |  |  |  |  |  |  | 22.47 ± 1.83 |  |  |  |  |  | 176.25±17.56 | 179.38±31.08 |  |
| Cai H et al.(2019) | TRE |  |  |  |  |  |  |  |  |  | 26.76±1.59 | 26.48±1.38 |  |  |  |  | 175.17±59.16 | 155.83±59.93 |  |
|  | CON |  |  |  |  |  |  |  |  |  | 26.34±2.73 | 26.48±1.60 |  |  |  |  | 188.7±53.36 | 172.08± 52.59 |  |
| Tinsley G M et al. (2019) | TRE |  |  |  | 113±2 | 111±2 |  | 67±1 | 66±2 |  |  |  |  |  |  |  | 179±10 | 172±11 |  |
|  | CON |  |  |  | 108±2 | 109±2 |  | 64±1 | 64±2 |  |  |  |  |  |  |  | 179±9 | 185±11 |  |
| Lowe D A et al.(2020) | TRE | 2.81*0.8 |  |  | 119.8 *3.7 |  |  | 76.9*3.8 |  |  | 32.9*4.9 |  |  | 5.28*0.14 |  |  | 203.7*14.5 |  |  |
|  | CON | 3.41*0.8 |  |  | 122.6*3.6 |  |  | 74.6*0.8 |  |  | 32.6*3.4 |  |  | 5.30*0.14 |  |  | 202.5*14.5 |  |  |
| Jamshed H et al. (2022) | TRE | 6.02*5.41 |  |  | 126*14 |  | -8*13.69 | 82*9 |  | -5*6.84 | 40.1*6.6 |  |  | 5.5*0.4 |  |  | 204*41 |  | -9*41.07 |
|  | CON | 4.44*2.88 |  |  | 122*11 |  | -3*13.69 | 80*8 |  | -1*6.84 | 39.2*6.8 |  |  | 5.5*0.4 |  |  | 202*36 |  | -15*41.07 |
| Liu D et al. (2022) | TRE |  | -1.4 *2.35 |  | 125.3±12.0 |  | -10.1*9.82 | 73.1±9.5 |  | -6.0*7.68 |  |  |  |  |  |  | 194.5±32.9 | -9.0*29.24 |  |
|  | CON |  | -1.2*2.56 |  | 124.8±12.2 |  | -8.1*9.39 | 74.5±9.6 |  | -5.1*7.47 |  |  |  |  |  |  | 198.0±37.8 | -13.7*27.74 |  |
| Xie Z et al.(2022) | TRE |  |  | -0.05±0.75 |  |  | 0.7±7.8 |  |  | -3.4±7.2 | 22.7*3.1 |  |  |  |  |  |  |  | -0.01 ± 0.57 |
|  | CON |  |  | -1.08±1.59 |  |  | -4.4±9.9 |  |  | 0.0±8.0 | 21.5*2.9 |  |  |  |  |  |  |  | 0.01 ± 0.43 |
| Haganes K L et al.(2022) | TRE |  | 2.2*0.8 |  | 121*10.7 | 118.7*11.5 |  | 79.9*9.0 | 78.6*10.1 |  | 31.8 *3.3 |  |  |  |  |  | 181.89*27.09 | 181.89*23.22 |  |
|  | CON |  | 2.4*1.2 |  | 122.4 *10.3 | 122.6*10.5 |  | 80.4*8.4 | 80.3*9.9 |  | 33.1*4.2 |  |  |  |  |  | 185.76*42.67 | 178.02*42.57 |  |
| Manoogian E et al.(2022) | TRE | 1.13*0.78 | 1.12*0.58 |  | 120.73*2.6 | 118.86*8.68 |  | 74.45 *2.2 | 72.78*9.04 |  | 27.77*0.8 | 27.52*3.80 |  | 5.3*0.4 | 5.27*0.28 |  |  |  |  |
|  | CON | 1.09*0.71 | 1.13 *0.75 |  | 120.32*2.2 | 119.72*9.04 |  | 74.21*2.1 | 72.45*9.03 |  | 27.65*1.1 | 27.56*2.4 |  | 5.28*0.31 | 5.29*0.33 |  |  |  |  |
| Che T et al.(2021) | TRE | 3.71±0.68 |  |  |  |  |  |  |  |  | 26.42±1.96 |  |  | 8.68±1.21 |  |  | 100.15± 47.17 |  |  |
|  | CON | 3.65±0.73 |  |  |  |  |  |  |  |  | 26.08±2.14 |  |  | 8.34±1.09 |  |  | 95.12±45.63 |  |  |
| Andriessen C et al.(2022) | TRE |  |  |  |  |  |  |  |  |  |  |  |  |  |  |  |  |  |  |
|  | CON |  |  |  |  |  |  |  |  |  |  |  |  | 6.4±0.7 |  |  |  |  |  |
| Thomas E A et al.(2021 | TRE |  |  |  |  |  |  |  |  |  | 34.6*5.8 |  |  | 6.4±0.7 |  | 0*0.18 |  |  | -5.55*18.25 |
|  | CON |  |  |  |  |  |  |  |  |  | 33.7 (5.6) |  |  |  |  | 0*0.15 |  |  | -4.56*4.56 |
| Phillips N.E et al.(2021) | TRE |  |  |  | 123.8*11.2 | 125.1*13.4 |  | 79.3*11.0 | 81.2*10.7 |  | 28.0*4.1 | 27.5*4.3 |  |  |  |  |  |  |  |
|  | CON |  |  |  | 126.4*10.5 | 122.3*13.5 |  | 81.0*9.6 | 79.5*11.0 |  | 27.0*4.0 | 26.7*4.0 |  |  |  |  |  |  |  |
| de Oliveira M P I et al.(2021) | TRE |  |  |  | 127.1±15.29 | 122.41*3.9 |  | 86.20±13.20 | 83.80*4.25 |  | 33.53±4.53 | 33.27*1.8 |  |  |  |  |  |  |  |
|  | CON |  |  |  | 124.03±11.28 | 119.44*4.0 |  | 86.51±10.08 | 82.29*4.3 |  | 33.12±3.63 | 32.96*1.8 |  |  |  |  |  |  |  |
| Rona A et al.(2018) | TRE |  |  |  |  |  |  |  |  |  | 29·0*1·7 |  |  |  |  |  | 178.22*16.23 |  |  |
|  | CON |  |  |  |  |  |  |  |  |  | 28·6*2·8 |  |  |  |  |  | 174.35*15.46 |  |  |
| Cienfuegos S et al.(2020) | (20:4)TRE |  |  |  | 134±4 | -5±2.2 |  | 88±2 | -2.8±1.0 |  | 37 ± 1 |  |  | 5.9±0.2 |  |  |  |  |  |
|  | (18:6)TRE |  |  |  | 130±4 | -4.4 ± 2.3 |  | 84±2 | -3.2±1.5 |  | 37 ± 1 |  |  | 5.9±0.1 |  |  |  |  |  |
|  | CON |  |  |  | 127±6 | 3.7±2.8 |  | 84±3 | 2.4±2.2 |  | 36 ± 1 |  |  | 6.0±0.2 |  |  |  |  |  |
| Mayra S T et al.(2022) | TRE |  |  |  |  |  |  | 68.6± 10.5 |  |  | 21.8±1.6 | 21.8±1.6 |  |  |  |  |  |  |  |
|  | CON |  |  |  |  |  |  | 69.6 ± 9.1 |  |  | 24.5±2.3 | 24.5±2.3 |  |  |  |  |  |  |  |
| Sutton E F et al. (2018) | TRE |  |  |  | 108.6±11.1 |  | -7.7*14.02 |  |  | -5.21*7.4 |  |  |  |  |  |  |  |  | -1.19*16.12 |
|  | CON |  |  |  | 111.1± 12.0 |  | 4.33*13.75 |  |  | 4.86*9.26 |  |  |  |  |  |  |  |  | -12.38*16.97 |
| Bei-ni Lao(2023) | TRE |  |  |  | 130.7士 11.3 | 130.9 +14.7 |  | 80.9土 6.0 | 79.7 士10.7 |  | 29.3±2.3 | 28.2 + 2.1 |  |  |  |  |  |  |  |
|  | CON |  |  |  | 125.4士 15.7 | 127.9+ 12.6 |  | 72.2士9.1 | 71.6+11.5 |  | 28.0 ±2.4 | 27.8 +2.3 |  |  |  |  |  |  |  |

**Table S4.** Ranks the likelihood of the measured results having the desired effect according to different eating windows

| Weight | | | | | |
| --- | --- | --- | --- | --- | --- |
| Control | <6h | 18:6 | 16:8 | 14:10 | =12 |
| 0.16222 | 0.79045 | 0.84181 | 0.46856 | 0.66322 | 0.07374 |
| Fasting insulin | | | | | |
| Control | <6h | 18:6 | 16:8 | 14:10 | =12 |
| 0.26 | 0.81 | 0.94 | 0.003 | 0.487 |  |
| HDL | | | | | |
| Control | <6h | 18:6 | 16:8 | 14:10 | =12 |
| 0.40254 | 0.12514 | 0.7064 | 0.477765 | 0.640253 | 0.647903 |
| LDL | | | | | |
| Control | <6h | 18:6 | 16:8 | 14:10 | =12 |
| 0.307338 | 0.659313 | 0.807213 | 0.364263 | 0.361875 |  |
| Fasting glucose | | | | | |
| Control | <6h | 18:6 | 16:8 | 14:10 | =12 |
| 0.261 | 0.8966 | 0.7214 | 0.359 | 0.0163 | 0.7457 |
| TG | | | | | |
| Control | <6h | 18:6 | 16:8 | 14:10 | =12 |
| 0.69585 | 0.71259 | 0.77073 | 0.42162 | 0.2313 | 0.16791 |


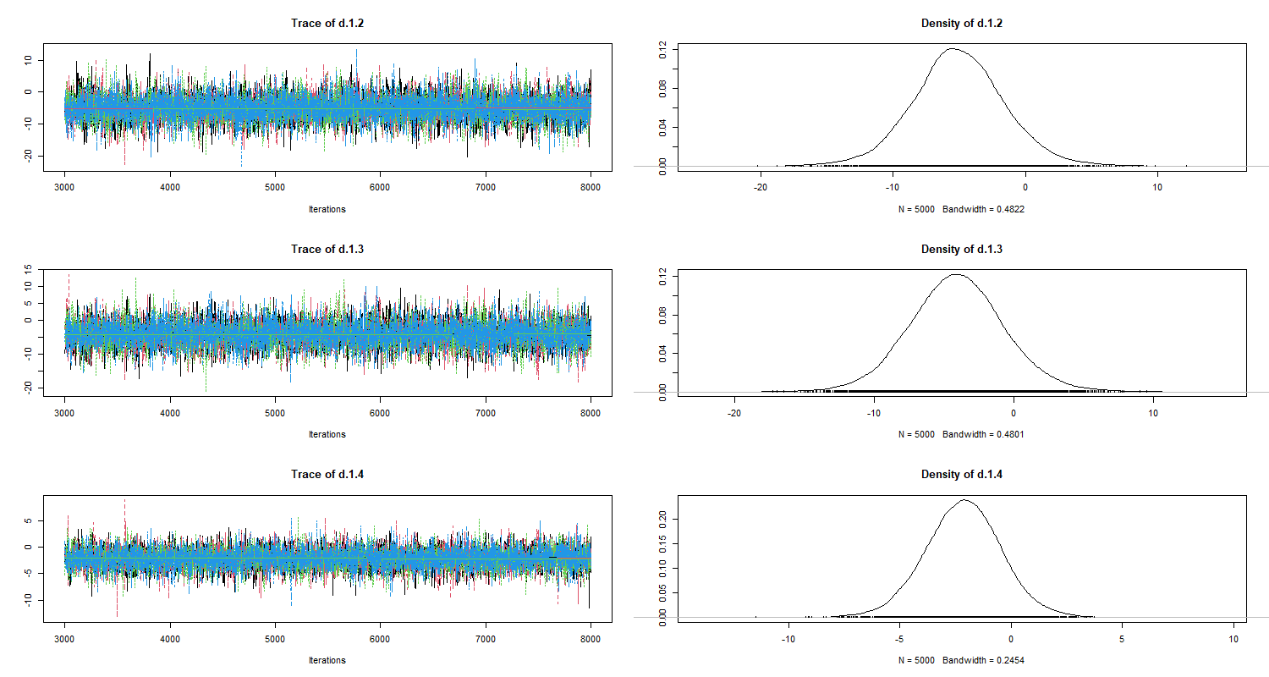


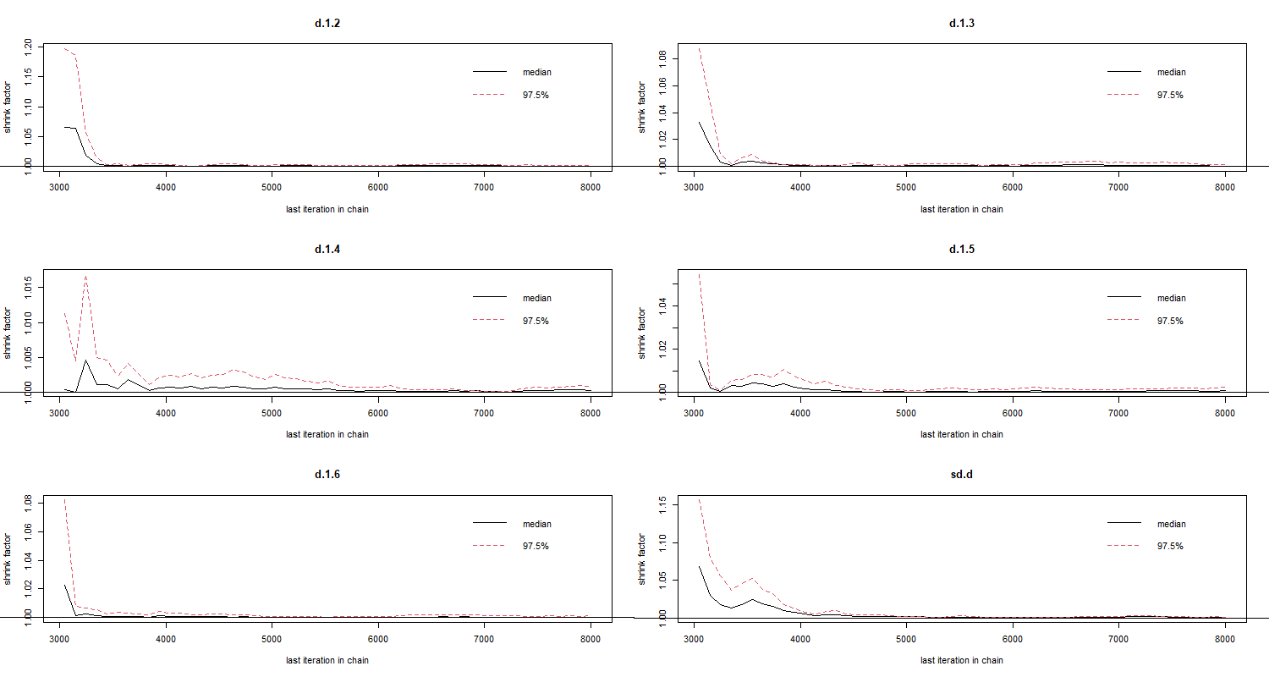


**Figure S1.** The convergence diagnostics of model and the Gelman-Rubin diagrams


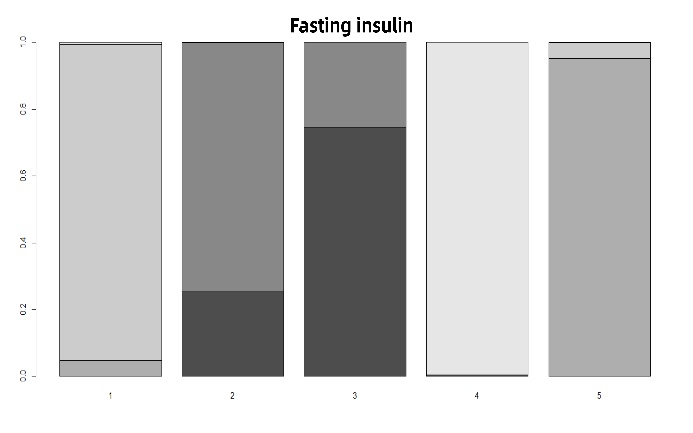

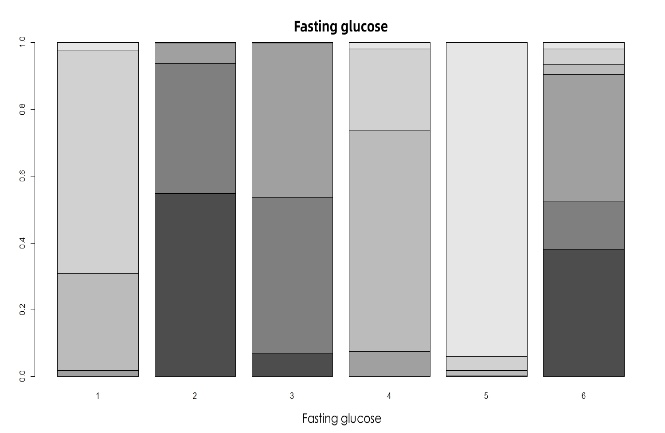

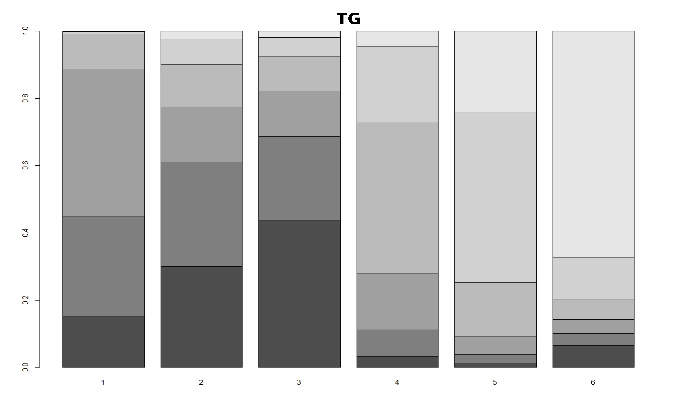

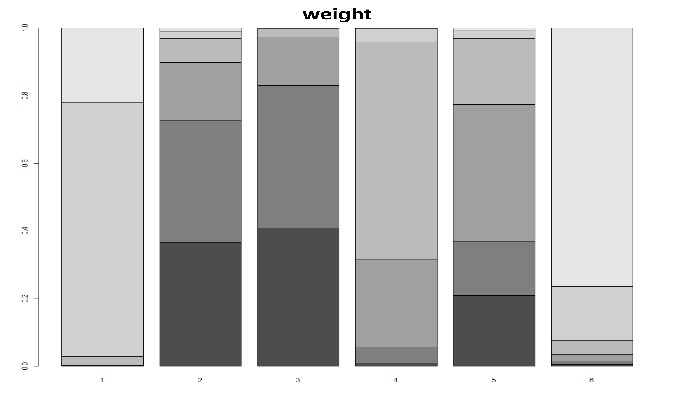


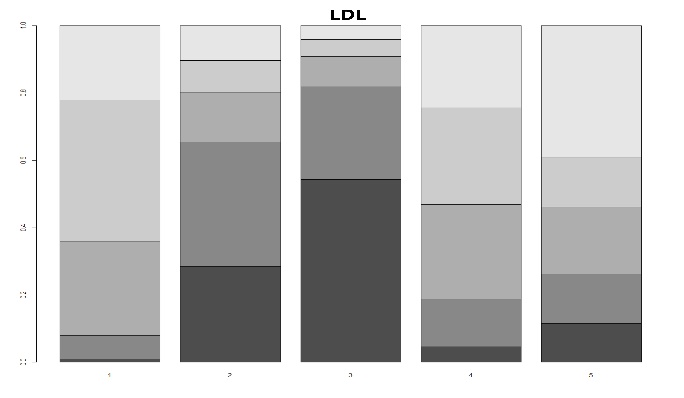


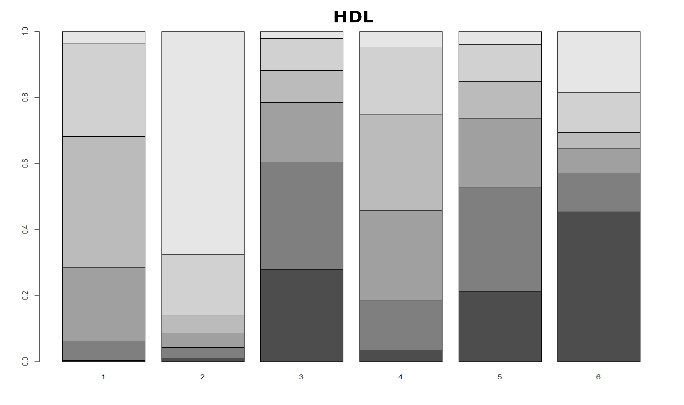


**Figure S2.** SUCRA graphes for Weight, Fasting insulin, HDL, LDL, TG, and Fasting glucose.

Note: 1: Control group; 2: ＜6 group; 3: 18:6 group; 4: 16:8 group; 5: 14:10 group; 6: =12 group.


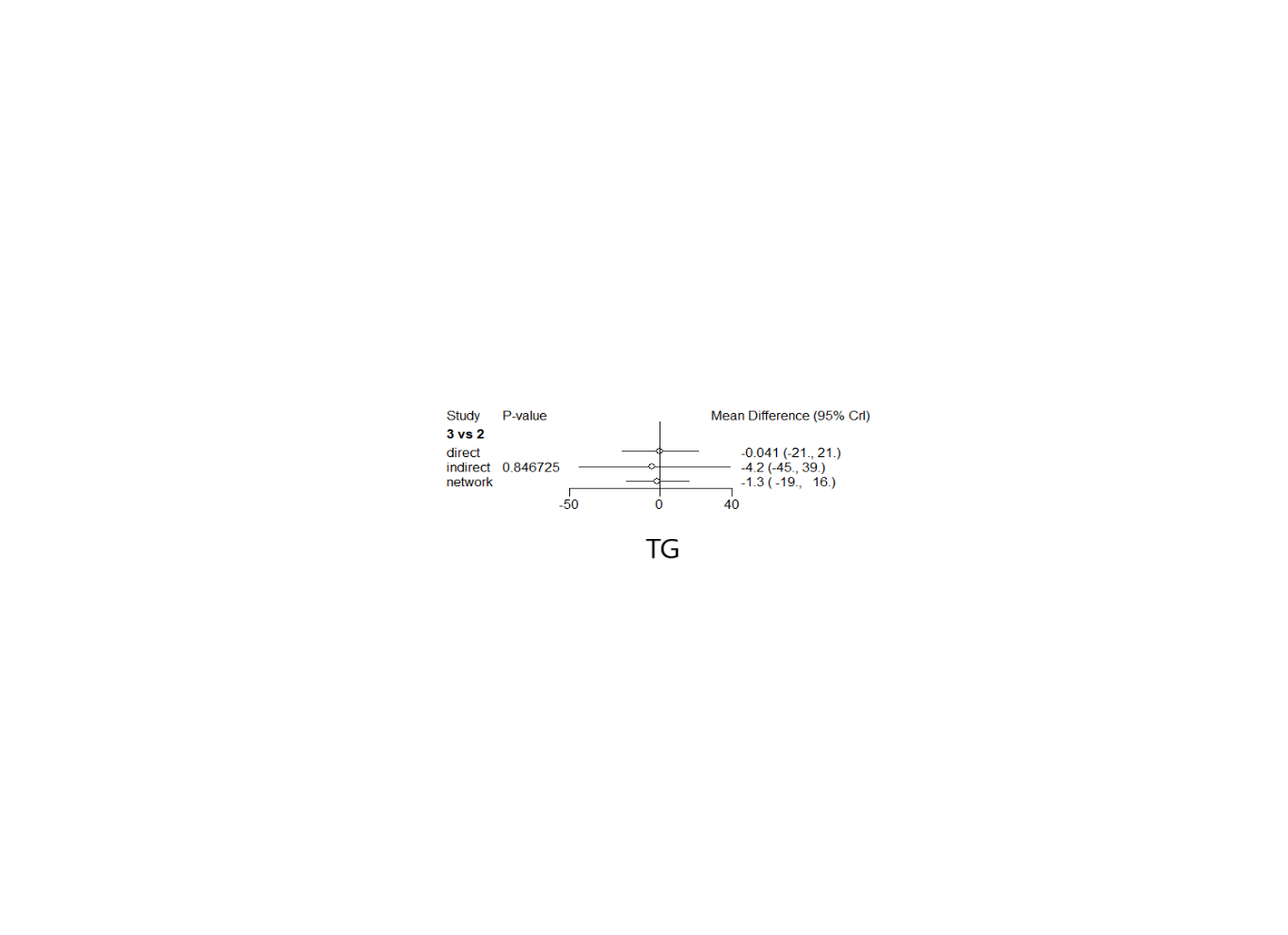

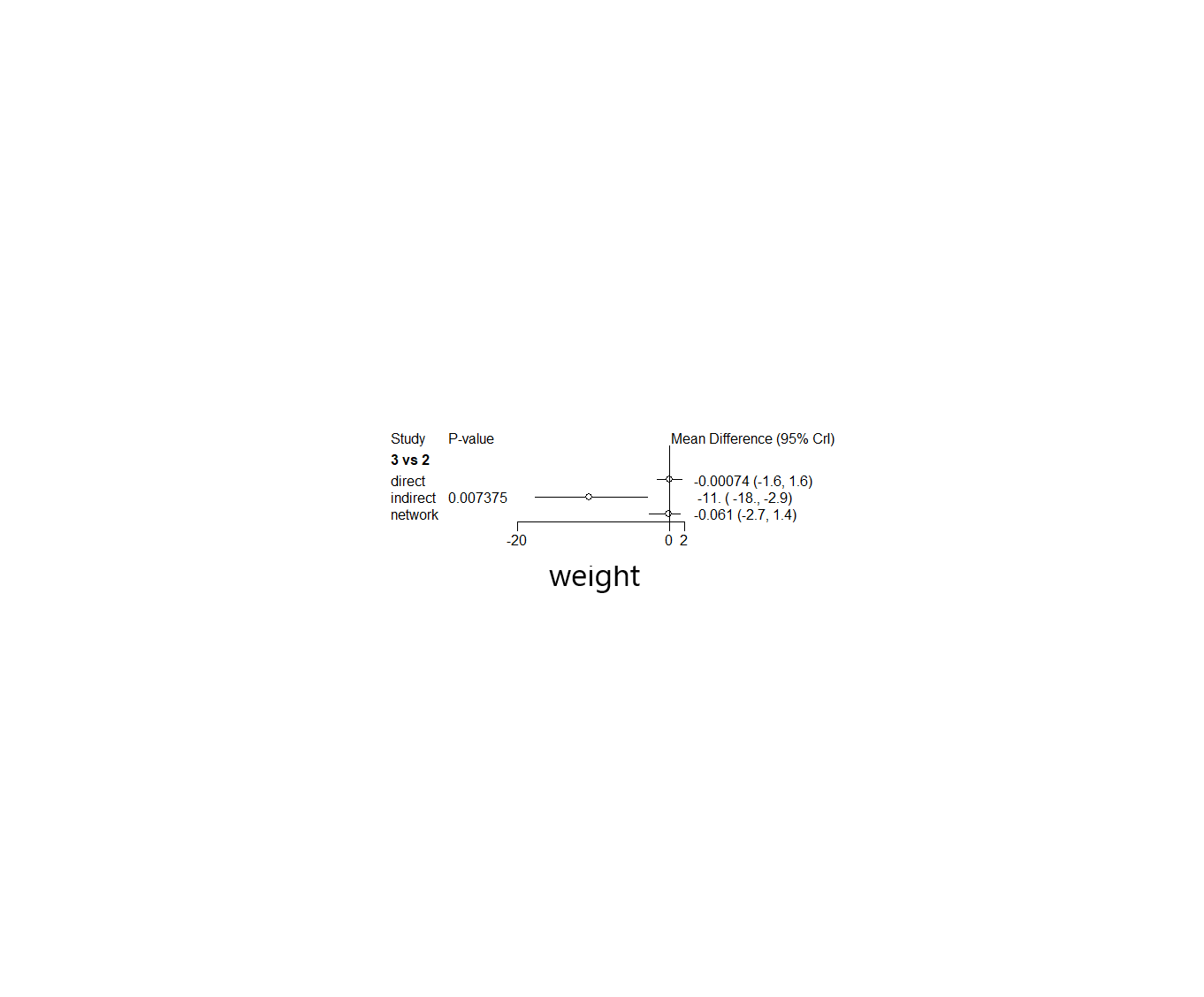

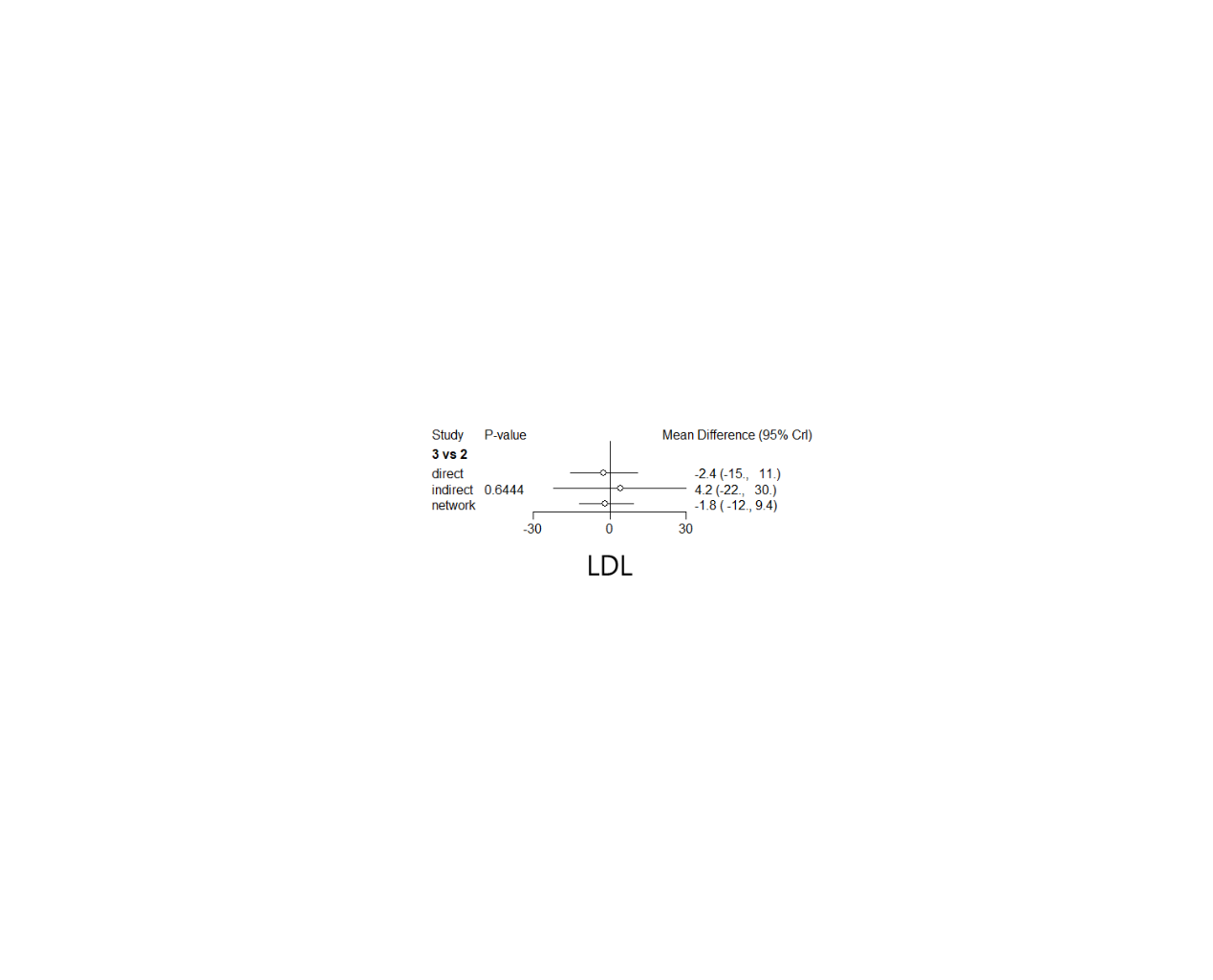

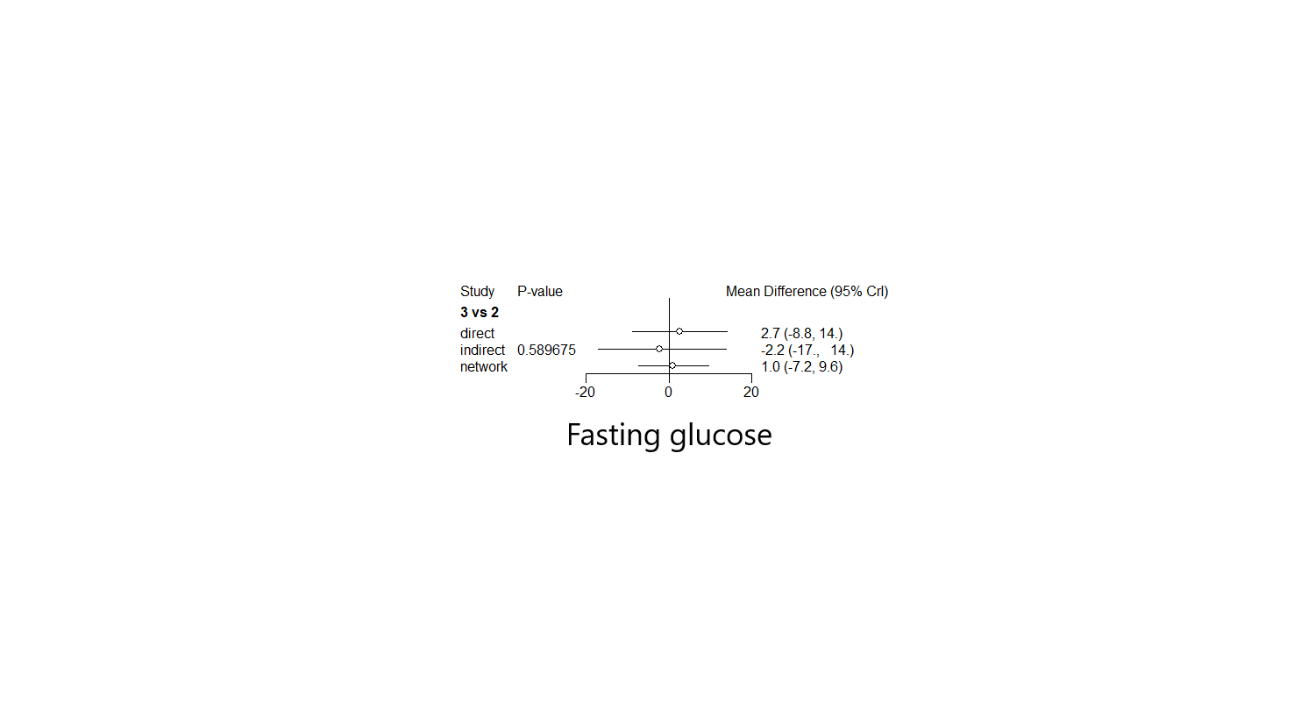


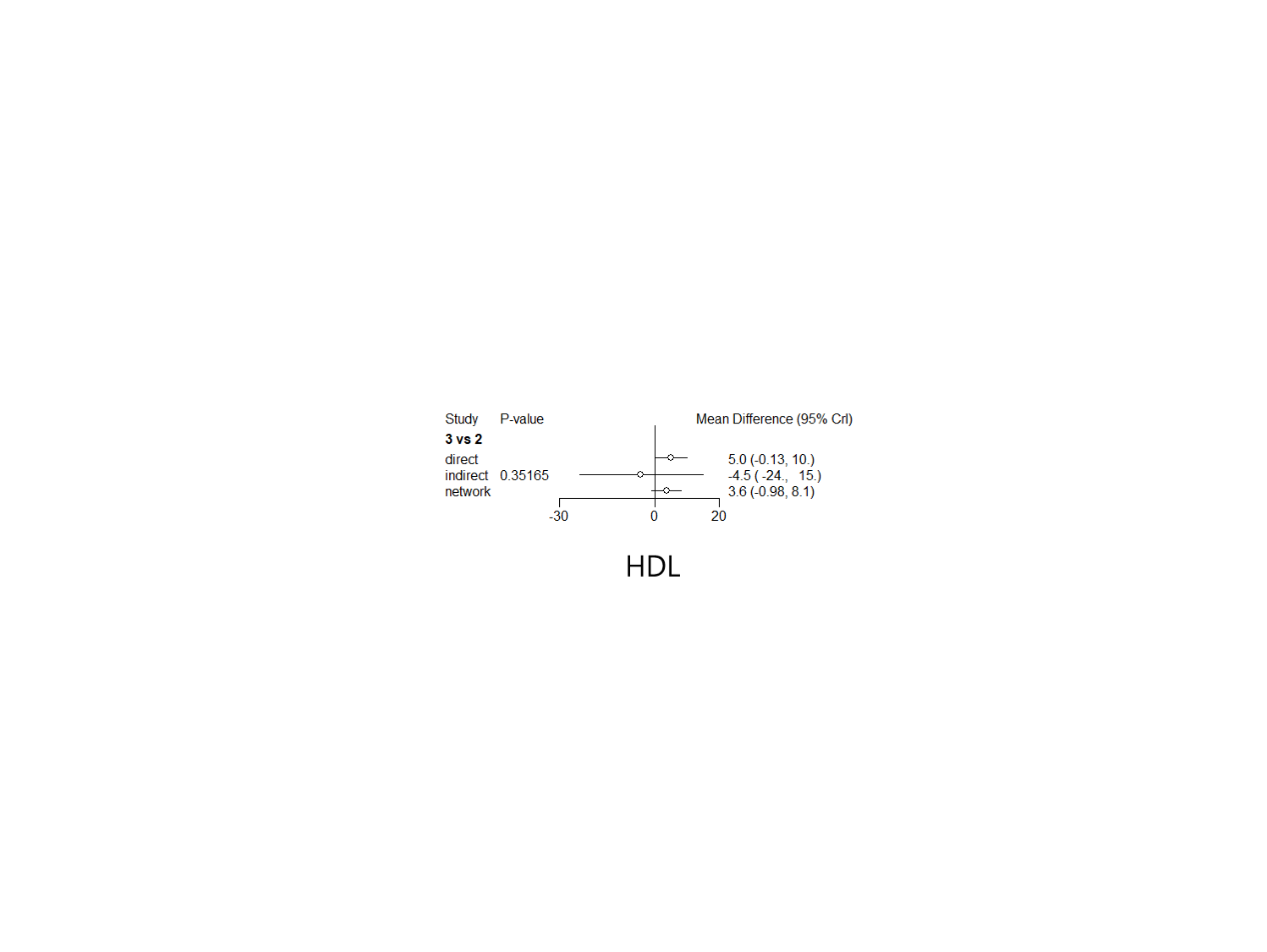


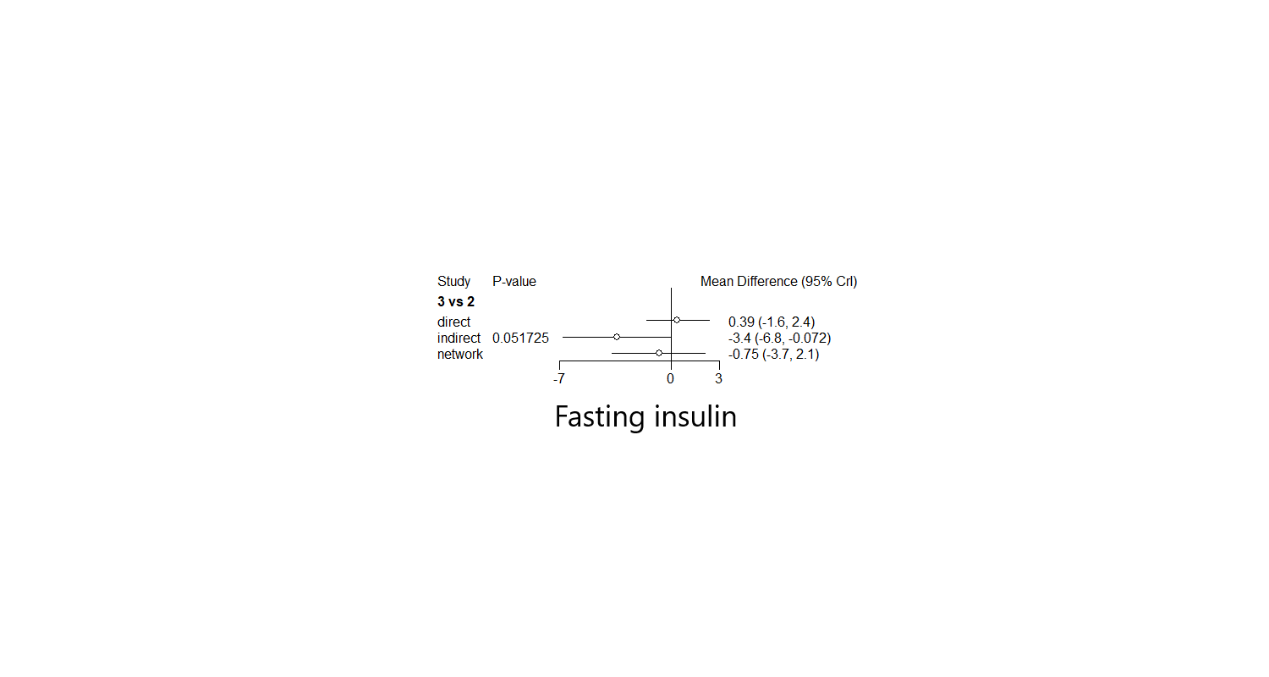


**Figure S3**. Inconsistency plots for all outcomes.

Note: 1: Control group; 2: ＜6 group; 3: 18:6 group; 4: 16:8 group; 5: 14:10 group; 6: =12 group.


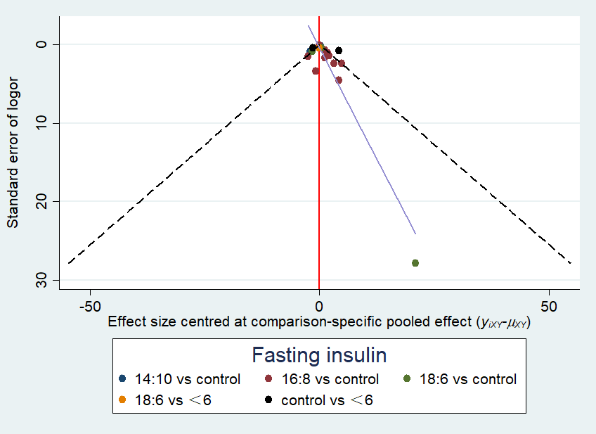

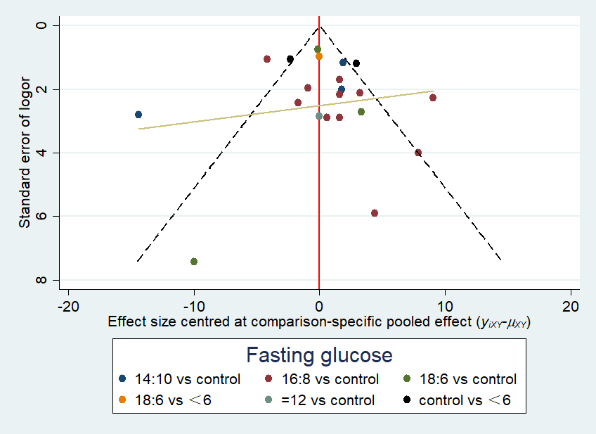

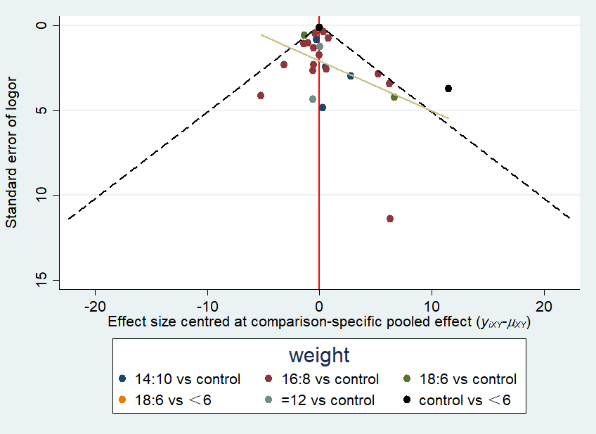

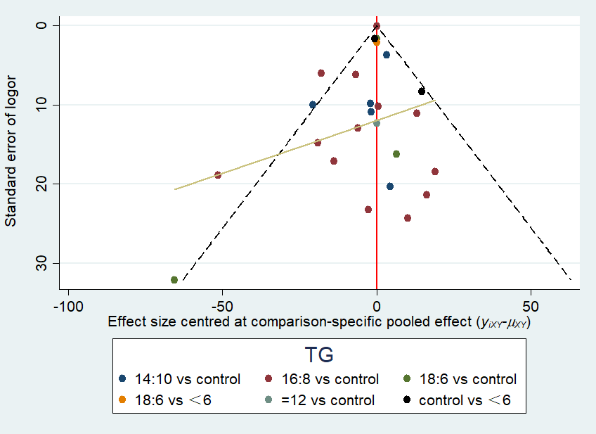

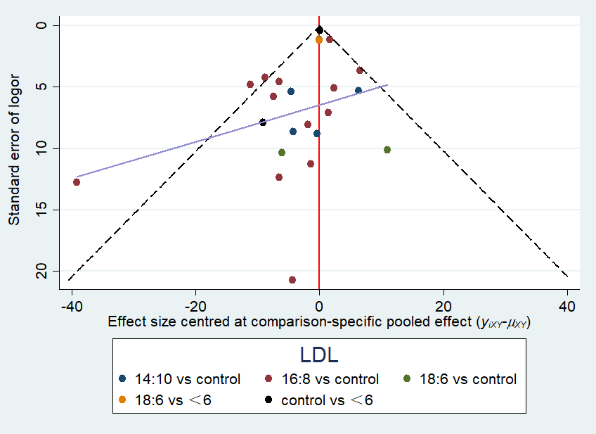

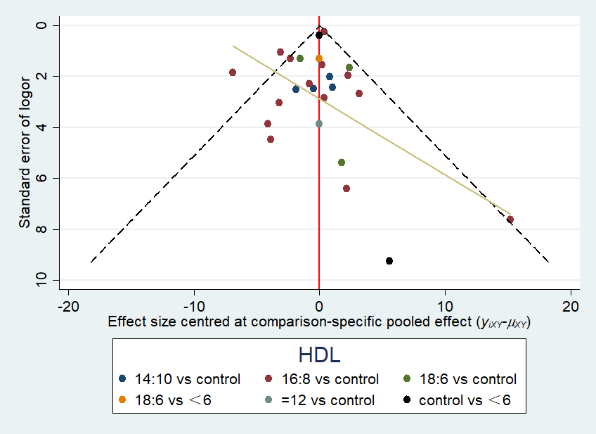


**Figure S4.** Comparison-adjusted funnel plot for all outcomes.
